# Structural and biochemical characterization of the *Cutibacterium acnes* exo-*β*-1,4-mannosidase that targets the *N*-glycan core of host glycoproteins

**DOI:** 10.1371/journal.pone.0204703

**Published:** 2018-09-27

**Authors:** Tom Reichenbach, Dayanand Kalyani, Rosaria Gandini, Olov Svartström, Henrik Aspeborg, Christina Divne

**Affiliations:** Department of Industrial Biotechnology, School of Engineering Sciences in Chemistry, Biotechnology, and Health (CBH), KTH Royal Institute of Technology, Stockholm, Sweden; Universidade Nova de Lisboa Instituto de Tecnologia Quimica e Biologica, PORTUGAL

## Abstract

Commensal and pathogenic bacteria have evolved efficient enzymatic pathways to feed on host carbohydrates, including protein-linked glycans. Most proteins of the human innate and adaptive immune system are glycoproteins where the glycan is critical for structural and functional integrity. Besides enabling nutrition, the degradation of host *N*-glycans serves as a means for bacteria to modulate the host’s immune system by for instance removing *N*-glycans on immunoglobulin G. The commensal bacterium *Cutibacterium acnes* is a gram-positive natural bacterial species of the human skin microbiota. Under certain circumstances, *C*. *acnes* can cause pathogenic conditions, acne vulgaris, which typically affects 80% of adolescents, and can become critical for immunosuppressed transplant patients. Others have shown that *C*. *acnes* can degrade certain host *O*-glycans, however, no degradation pathway for host *N*-glycans has been proposed. To investigate this, we scanned the *C*. *acnes* genome and were able to identify a set of gene candidates consistent with a cytoplasmic *N*-glycan-degradation pathway of the canonical eukaryotic *N*-glycan core. We also found additional gene sequences containing secretion signals that are possible candidates for initial trimming on the extracellular side. Furthermore, one of the identified gene products of the cytoplasmic pathway, AEE72695, was produced and characterized, and found to be a functional, dimeric exo-*β*-1,4-mannosidase with activity on the *β*-1,4 glycosidic bond between the second *N*-acetylglucosamine and the first mannose residue in the canonical eukaryotic *N*-glycan core. These findings corroborate our model of the cytoplasmic part of a *C*. *acnes N*-glycan degradation pathway.

## Introduction

**Fig 1:**
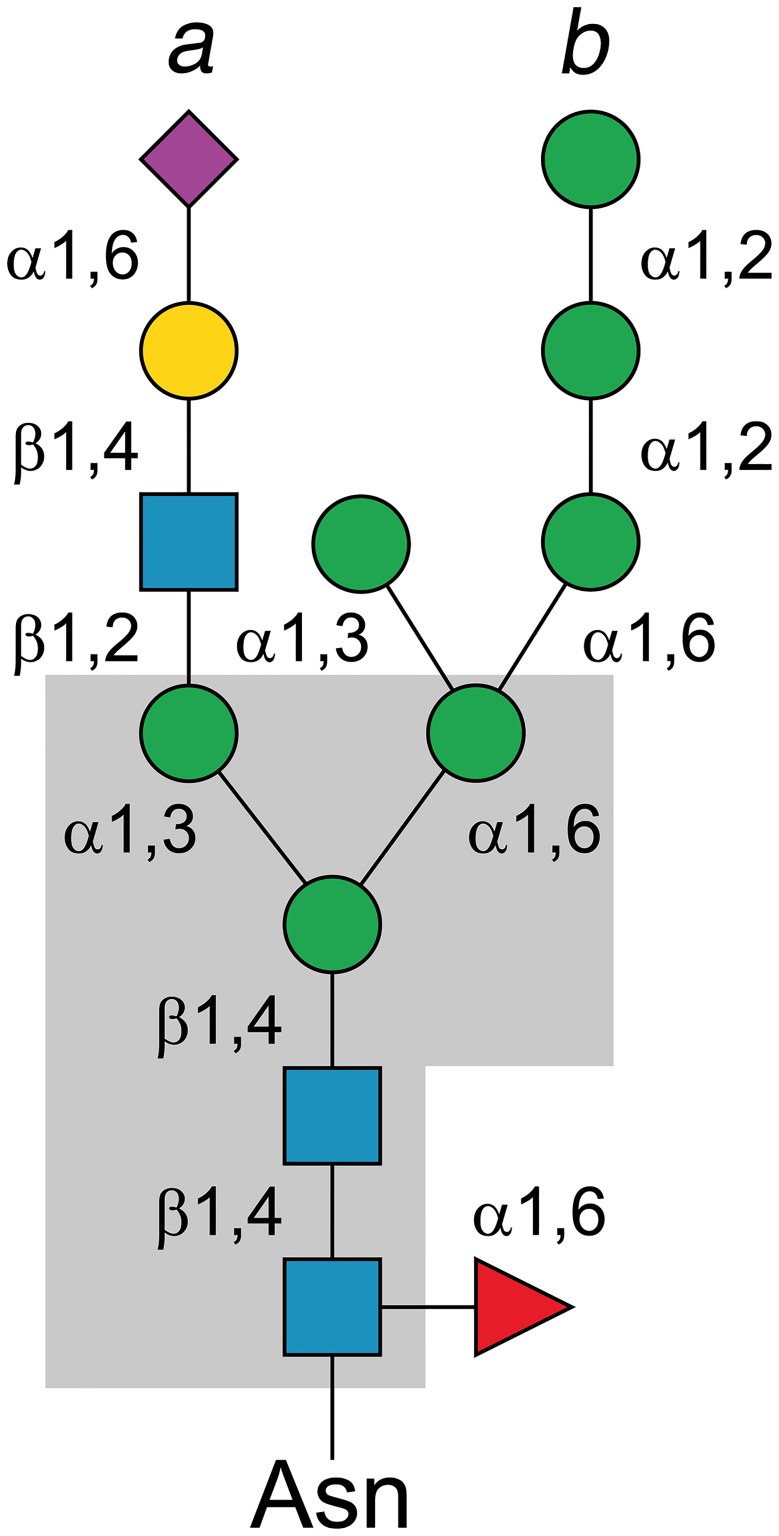
Schematic structure of an *N*-glycan. The *N*-glycans contain different carbohydrates depending on the type, but share a common core structure (gray box): branch *a*, complex *N*-glycan consisting of two to four branches with mixed sugar residues; branch *b*, high-mannose *N*-glycan contains only mannose residues beyond the core structure; mixed *a*+*b*, a combination of complex and high-mannose type *N*-glycan. Color scheme: blue square, *N*-acetylglucosamine; green circle, mannose; yellow circle, galactose; purple diamond, sialic acid; red triangle, fucose.

More than two-thirds of all human proteins are predicted to be glycosylated [1], which makes glycosylation one of the most common post-translational protein modifications [2,3]. Protein glycosylation can alter the biophysical properties and function of native proteins that are coded by the genomic sequence [3,4], as for example in the case of proteins of the immune system, *e*.*g*., immunoglobulins, where *N*-glycosylation offers structural and functional diversity beyond that provided by genetic V(D)J recombination [5,6]. In eukaryotes, the *N*-glycan precursors will be step-wise assembled at the endoplasmic reticulum (ER) through the successive transfer of sugar units (glucose, Glc; mannose, Man; N-acetylglucosamine, GlcNAc) catalyzed by glycosyltransferases [4,7]. The assembled precursor will be transferred “en bloc” to a protein asparagine (present in the sequon Asn-X-Thr/Ser) by the enzyme oligosaccharyltransferase (OST) in the ER lumen [8–10]. All three known *N*-glycans: high-mannose, complex and hybrid, share an identical five-sugar core structure with the sequence Man_3_-GlcNAc_2_ (Fig 1) [11].

Considering the magnitude of the human glycoproteome, and the importance of simple sugars as nutrients for microorganisms, it is not surprising that commensal and pathogenic bacteria have developed efficient systems to degrade and utilize the abundant sugar pool offered by protein-linked glycans [12,13]. By degrading immunoglobulin-linked *N*-glycans, a bacterial pathogen can alter the host’s immune response to its best advantage [14–20]. For example, in the case of the pathogen *Streptococcus pneumoniae*, efficient virulence requires an endo-*β*-*N*-acetylglucosaminidase that hydrolyzes the *β*-1,4-glycosidic bond between the two GlcNAc residues in the *N*,*N*-diacetylchitobiose (GlcNAc_2_) core, as well as an *α*-1,2-mannosidase to trim the terminal *α*-1,2-linked mannose residues of high-mannose *N*-glycans down to the Man_3_-GlcNAc core [21].

The gram-positive bacterium *Cutibacterium acnes* (formerly *Propionibacterium acnes*) belongs to the phylum Actinobacteria, and is one of the major inhabitants of the sebaceous follicles of the human skin. Some individuals develop acne vulgaris, *i*.*e*., a typically chronic, inflammation of the pilosebaceous unit resulting from *C*. *acnes* colonization of the sebaceous glands connected to the hair follicles in the pilosebaceous unit. The disease is multifactorial, however, the androgen-mediated increase in sebum production in adolescents and young adults in combination with *C*. *acnes* colonization of the sebaceous glands is a typical disease trigger. Furthermore, *C*. *acnes* can become particularly problematic in immunosuppressed transplant patients where 20–25% are affected by acne infections. Recent results from genome-wide association studies have also shown that complex patterns of genetic predisposing factors can trigger pathological acne infection [22–24].

*C*. *acnes* has been shown to degrade *O*-linked glycans, and there are indications that the bacterium can cleave off sugar components from glycolipids [25–27]. Although the first sequenced *C*. *acnes* genome was released in 2004, and the current number of available *C*. *acnes* genomes is close to a hundred [28,29], there are thus far no reports of attempts to identify *N*-glycan-degrading enzymes. With the aim to arrive at a more detailed picture of the host-microbe interactions associated with *C*. *acnes* in general, and clinically relevant acne infection in particular, we have, to this end, analyzed the *C*. *acnes* genome for genes coding for glycoside hydrolases (GHs) that are candidate enzymes in an *N*-glycan-degradation (NGD) pathway. In this work, we propose a degradation pathway for the cytoplasmic path of host *N*-glycans by *C*. *acnes*, as well as a detailed structural and functional characterization of the exo-*β*-1,4-mannosidase, a member of glycoside hydrolase family 5 subfamily 18 (GH5_18), that catalyzes hydrolysis of the *β*-1,4-glycosidic bond between the second *N*-acetylglucosamine and the first mannose residue in the canonical eukaryotic *N*-glycan core.

## Material and methods

### Identification of candidate genes for *N*-glycan degradation

The genome analyses were based on data available in the carbohydrate-active enzymes (CAZy) database (www.cazy.org) [30], the *C*. *acnes* strain 266 genome (CP002409) [31], as well as other *C*. *acnes* genomes publicly available in the NCBI database. Operons were investigated using MicrobesOnline Operon Predictions [32]. One unique gene, AEE72695, coding for an enzyme classified in CAZy as belonging to family 5, subfamily 18 (GH5_18) was selected for a more detailed phylogenetic analysis, and we hereafter refer to this gene product as *Ca*Man5_18. Sequences belonging to GH5_18 were retrieved from CAZy, or identified using the Basic Local Alignment Search Tool (BLAST). A total of 55 sequences that represented the diversity of the subfamily were trimmed and used as input for multiple amino-acid sequence alignment using MUSCLE [33]. PhyML was used to create a maximum likelihood tree [34] available at (http://www.phylogeny.fr) [35]. The statistical significance of the branches was assessed with the Approximate Likelihood-Ratio Test (aLRT). The phylogenetic tree was displayed using the Interactive Tree Of Life (iTOL) v3. The presence of possible signal peptides was analyzed using SignaIP 4.1 [36]. ESPript 3 was used to display the sequence alignment (http://espript.ibcp.fr/ESPript/cgi-bin/ESPript.cgi) [37] using pre-aligned sequences from Clustal X.

### Cloning and site-directed mutagenesis of *Ca*Man5_18

The gene (AEE72695) coding for *Ca*Man5_18 from *Cutibacterium acnes* strain 266 was synthesized and codon-optimized for expression in *Escherichia coli* by Gen9 Inc. The gene was subcloned into the ligation-independent cloning (LIC) vector pNIC-CH2 adding a C-terminal, non-cleavable hexa-histidine tag (https://ki.se/en/mbb/protein-production-platform) using the following forward and reverse primers:

LIC_fwd: 5’-AGAGGAGATAATTAATGAAGATTGGCGCCAATTACAC-3’
LIC_rev: 5’-AATGGTGGTGATGATGGTGCGCGTGGGCTCGAGCACTGG-3’

Site-directed mutagenesis to generate a catalytically deficient *Ca*Man5_18 mutant (E140Q/E259Q) was performed using the following forward and reverse primers:

E140Q_fwd (5’-ATGACGGTGGGCAACCAGTTCCCGCAGTACGCA-3’)
E140Q_rev (5’-TGCGTACTGCGGGAACTGGTTGCCCACCGTCAT-3’)
E259Q_fwd (5’-CCACTCTGGCTGCAGCAGGTAGGAGCACCTCGA-3’)
E259Q_rev (5’-TCGAGGTGCTCCTACCTGCTGCAGCCAGAGTGG-3’)

The PCR samples contained 50 ng plasmid DNA, 2 U Phusion High-Fidelity DNA polymerase (Thermo Fisher), 230 nM of each primer, 500 μM of each dNTP, and 1 x HF Phusion buffer (Thermo Fisher). For mutagenic PCR the following conditions were used: 95°C for 30 s, 30 cycles of 95°C for 30 s; 68°C for 9 min, with a final incubation at 68°C for 10 min. The resulting PCR products were treated with 10 U of DpnI (Thermo Fisher) to degrade the methylated template-DNA. The remaining PCR products were purified using the GeneJET PCR Purification Kit (Thermo Fisher), and transformed into chemically competent *E*. *coli* DH5*α* cells (Invitrogen) followed by plating on Luria-Bertani (LB) agar supplemented with 50 μg mL^-1^ kanamycin at 37°C for 17 h.

### Production of recombinant *Ca*Man5_18

The recombinant plasmid was transformed into *E*. *coli* BL21(DE3)-T1 competent cells. The bacteria were cultured in Terrific Broth (TB) medium supplemented with 50 μg mL^-1^ kanamycin. After reaching an OD_600_ value in the range 0.8 to 1.0, recombinant gene expression was induced by adding *β*-D-1-thiogalactopyranoside (IPTG) to a final concentration of 0.2 mM, and the culture further incubated at 17°C for 16 h. The cells were harvested by centrifugation at 5,000 r.p.m. (Beckman Coulter JA-10 fixed-angle rotor, 4,424×*g*). The bacterial pellet was resuspended in 25 mM K_2_HPO_4_ (pH 7.2), 150 mM NaCl, 5% (v/v) glycerol, with one tablet of complete protease inhibitor cocktail (Roche). The resuspended pellet was homogenized using an AVESTIN Emulsiflex-C3 system, and centrifuged at 10,000 r.p.m. (Beckman Coulter JA-25.50 fixed-angle rotor, 12,096×*g*) using an Avanti J20XP centrifuge (Beckman Coulter) for 20 min at 4°C. The resulting supernatant was mixed with Ni-NTA agarose resin (Invitrogen), packed in Bio-Rad Econo-Pac columns, and set aside to incubate at 4°C for 1 h. The columns were washed with 25 mM K_2_HPO_4_ (pH 7.2), 150–300 mM NaCl, 30–50 mM imidazole, 0–10% (v/v) glycerol. Bound protein was eluted with buffer containing 500 mM imidazole. The eluted protein was concentrated using Vivaspin20 centrifugal concentrators (polyethersulfone filter; molecular weight cut-off, MWCO, 30 kDa), and loaded onto a HiLoad 16/60 Superdex 200 prep grade column (GE Healthcare Life Sciences) equilibrated with 50 mM 4-(2-hydroxyethyl)-1-piperazineethanesulfonic acid (HEPES) pH 7.5, 150 mM NaCl, and 0–10% (v/v) glycerol. The recovered fractions containing *Ca*Man5_18 were pooled and further concentrated to 15–20 mg L^-1^ using a Vivaspin20 centrifugal spin concentrator (MWCO, 30 kDa). The same procedure as described above for the wild type was used for the E140Q/E259Q mutant.

### SEC analysis of oligomeric state

A size-exclusion chromatography (SEC) HiLoad 16/60 Superdex 200 prep grade column (GE Healthcare Life Sciences) was equilibrated in 25 mM K_2_HPO_4_ (pH 7.2), 150 mM NaCl, and calibrated with five protein standards: ferritin (440,000 Da), aldolase (158,000 Da), conalbumin (75,000 Da), ovalbumin (43,000 Da) and ribonuclease (13,700 Da); after which *Ca*Man5_18 was subjected to SEC to determine the retention volume. The logarithm of the molecular weights (log_10_MW) of the protein standards were plotted *versus* the retention volume using GraphPad Prism 7 (GraphPad Software, San Diego California USA) to obtain a standard curve (*R*^2^ = 0.9974) for extrapolation of the log_10_MW for *Ca*Man5_18.

### Structure determination and model refinement

Crystals of *Ca*Man5_18 were grown by vapor diffusion in sitting drops at 4°C in the presence of metal ion and substrate. Successful crystallization was achieved by mixing 1 μL 25 mg mL^-1^ protein in 20 mM HEPES pH 7.5, 300 mM NaCl, 10% (v/v) glycerol, 0.5 mM tris(2-carboxyethyl)phosphine (TCEP) with 0.5 μL reservoir solution containing 0.2 M 3-(*N*-morpholino) propane sulfonic acid (MOPS) buffer (pH 6.5), 0.2 M magnesium acetate, 20% (w/v) polyethylene glycol (PEG) 8000 and 5 mM D-mannose. For experimental phasing, a solution of gold cyanide, Au(CN)_2_, was added to a final concentration of 10 mM in the drop.

X-ray intensity data for non-derivatized and derivatized crystals were recorded using synchrotron radiation, followed by data merging and scaling using the *XDS* package [38]. The crystals belong to space group *P*2_1_ with four molecules in the asymmetric unit, and an approximate solvent content of 50%. Heavy-atom substructure determination and initial phasing were performed on data from the gold-derivatized crystal by single-wavelength anomalous dispersion (Au-SAD) as implemented in the program *autoSHARP* [39] included in the *SHARP* package [40]. The initial SAD phases were improved and extended to 1.8 Å resolution by solvent flattening using *SOLOMON* as implemented in *SHARP*.

An initial model for *Ca*Man5_18 was built by alternating manual model building using COOT [41], and refinement with *PHENIX* [42] guided by *σ*_A_-weighted 2*F*_o_-*F*_c_ electron density maps. Refinement with *PHENIX* included refinement of x,y,z coordinates, real-space refinement, and refinement of individual atomic displacement parameters. The gold contribution to the Fourier amplitudes induced undesirable effects in the electron density near the sites of substitution, which obstructed map interpretation and completion of the model in areas close to the heavy-atom sites. Calculated phases from the partial model were therefore used to phase the Fourier amplitudes from a native, non-derivatized crystal. The resulting 2*F*_o_-*F*_c_ electron density map was of high quality and a complete *Ca*Man5_18 model was generated. Refinement statistics are given in Table 1.

**Table 1:** Data collection, phasing and refinement statistics.

*Data collection*	*Ca*Man5_18	*Ca*Man5_18 derivative
Protein variant	Native	Au(CN)_2_
Cell constants a, b, c (Å); β(°)	84.18, 104.37, 112.75; 90.27	83.65, 101.44, 111.86; 90.13
Space group / molecules per a.s.u.	*P*2_1_ / 4	*P*2_1_ / 4
Beamline, λ (Å)	SOLEIL PROXIMA1, 1.07175	SOLEIL PROXIMA1, 1.03927
Resolution range, nominal (Å)	49.60–1.70 (1.80–1.70)	48.98–2.00 (2.10–2.00)
Unique reflections	207,504 (31,843)	217,603 (16,499)
Multiplicity	6.8 (6.9)	3.3 (2.9)
Completeness (%)	97.1 (94.9)	87.5 (48.7)
<*I* / σ*I*>	8.8 (0.9)	10.9 (1.69)
*R*_sym_	0.111 (2.74)	0.076 (0.692)
*R*_meas_	0.121 (2.96)	0.090 (0.846)
*CC*(1/2)	99.8 (44.0)	99.7 (62.9)
*CC*(1/2)_ano_	N/A	0.39 (0.12)
Wilson *B* factor (Å^2^)	29.3	25.9
*SAD phasing*		
Resolution (Å)		2.00
Number of sites		17
PP_ano_ (acen)		0.773
Rcullis_ano_ (acen)		0.889
FOM (acen/cen)		0.248/0.082
Solomon E^2^/contrast (solvfrac)		2.1895 (0.506)
*Crystallographic refinement*		
Resolution range (Å)	49.601–1.800 (1.845–1.800)	
Completeness, all % (outer bin)	97.5 (96.0)	
*R*_*factor*_/work reflns, all	0.195 / 175,603	
*R*_*free*_/free reflns, all	0.226 / 2001	
Number of amino-acid residues	1,575	
Non-hydrogen atoms	13,584	
Mean *B* (Å^2^) protein all	33.5 / 12,748	
Mean *B* (Å^2^) solvent / N°. mol.	37.2 / 836	
Rmsd bond lengths (Å), angles (°)	0.007, 0.86	
Ramachandran: favored (%) / allowed (%) / Outliers	97.8 / 100 / 0	
PDB accession code	6GVB	

### ThermoFluor stability assay

Thermal stability to unfolding of *Ca*Man5_18 was analyzed using ThermoFluor screening [43]. The effect of pH on the melting temperature, *T*_m_, was investigated. Reaction mixtures of 100 μL contained 2 μL of 10 mg mL^-1^ *Ca*Man5_18, 2 μL SYPRO Orange dye (25x stock solution diluted 1:200 with water), and 97 μL 50 mM buffer (sodium acetate pH 5.0–5.5, sodium phosphate pH 6.0–7.5, and Tris-HCl pH 8.5–9.0), and were added to the wells of a 96-well thin-wall PCR plate (Bio-Rad). The pH value was varied from 5 to 9 in intervals of 0.5 pH units. The plates were sealed with Optical-Quality Sealing Tape (Bio-Rad), and heated in an iCycler iQ Real Time PCR Detection System (Bio-Rad) from 20–90°C in steps of 0.2°C. The changes in fluorescence were monitored with a charge-coupled device (CCD) camera. The wavelengths for excitation and emission were 490 nm and 575 nm, respectively.

### Substrate screening

The substrate specificity of *Ca*Man5_18 was investigated by screening a range of *p*-nitrophenyl *β*-D-glycosides and polysaccharides: *p*-nitrophenyl-*β*-D-mannopyranoside (*p*NP-*β*Man), *p*-nitrophenyl-*β*-D-xylopyranoside (*p*NP-*β*Xyl), *p*-nitrophenyl-*β*-D-fucopyranoside (*p*NP-*β*Fuc), *p*-nitrophenyl-*α*-L-fucopyranoside (*p*NP-*α*Fuc), *p*-nitrophenyl-*β*-D-galactopyranoside (*p*NP-*β*Gal), *p*-nitrophenyl-*β*-D-glucopyranoside (*p*NP-*β*Glc), and *p*-nitrophenyl-*β*-D-cellobioside (*p*NP-*β*Cel) were purchased from Sigma Chemical Co. (St. Louis, MO); ivory nut mannan, konjac glucomannan, birchwood xylan, barley *β*-glucan, carboxymethylcellulose (CMC, low viscosity), and cellobiose were purchased from Megazyme (Bray, Ireland).

The enzyme reactions were performed in final volumes of 210 μL. The reaction mixtures contained 200 μL 1 mM *p*NP sugar in 50 mM sodium phosphate buffer (pH 6.0) and were pre-heated at 50°C for 5 min before adding 10 μL 10 mg/mL *Ca*Man5_18. Following incubation at 50°C for 10 min, 20 μL 2 M Na_2_CO_3_ was added to inactivate the enzyme and stabilize the chromophore in its anionic 4-nitrophenolate form. The released *p*-nitrophenol was measured spectrophotometrically at 410 nm. The amount of *p*NP released was measured at A_410_ (ε_410_ = 18.3 mM^-1^ cm^-1^). For cellobiose, CMC, glucomannan, mannan, barley *β*-glucan and xylan, 1% substrate was used, and the reaction was stopped by heating the sample at 100°C for 5 min and liberated glucose was measured by the dinitrosalicylic (DNS) colorimetric method. The data were analyzed with SigmaPlot (Systat Software, San Jose, CA).

### Kinetic analysis with *p*NP-*β*Man as substrate

The enzyme kinetics was determined for hydrolysis of *p*NP-*β*Man at various substrate concentrations (0.5 to 20 mM). The enzyme reaction was performed as described above for *p*NP sugars. Enzyme and substrate-free controls were prepared and assayed under the same conditions. One unit of enzyme activity was defined as the amount of enzyme required to release 1 μmol of *p*NP min^-1^ mL^-1^ under the standard assay conditions. The data were analyzed with GraphPad Prism 7 (GraphPad Software, San Diego California USA). All enzyme assays were performed in triplicates.

### Effect of pH and temperature on enzyme activity on *p*NP-*β*Man

The optimal pH (pH_opt_) and temperature (*T*_opt_) for enzyme activity were investigated by incubating 10 μg of *Ca*Man5_18 with *p*NP-*β*Man at 50°C for 10 min. For the determination of optimal pH for enzyme activity, the pH range 4.0 to 8.5 (in intervals of 0.5) was analyzed using the following buffer systems: 50 mM sodium acetate, pH 4.0 to 5.5; 50 mM sodium phosphate, pH 6.0 to 7.5; 50 mM Tris-HCl, pH 8.0 to 8.5. For determination of optimal reaction temperature, the enzyme activity was measured at pH 6.0 for the temperature range 20–80°C (in intervals of 10°C). The activity obtained at the *T*_opt_ or pH_opt_ was used to calculate the relative percentage of enzyme activity at different temperature and pH values.

The kinetic stability was determined by incubating 10 μg of *Ca*Man5_18 at 40°C, 50°C, 60°C and 70°C for 0 to 40 h (samples were taken at the time points: 0, 5, 10, 20, 30, 40, 60, 80 min; and 2, 4, 6, 12, 24, 36, 40 h). Following incubation, residual activity was immediately measured for the samples, and the half-life time of irreversible thermal inactivation (*a*_*t*_) was determined by non-linear regression using the equation of exponential decay:

$$a_{t}={a_{0}}^{\left(-t*ln\frac{2}{T_{1/2}}\right)}$$

Data were collected in triplicate, and analyzed using SigmaPlot (Systat Software, San Jose, CA).

### TLC analysis of reaction products

The products resulting from *Ca*Man5_18 hydrolysis of mannooligosaccharides, and the candidate for natural substrate, Man-GlcNAc, were analyzed by thin-layer chromatography (TLC). Briefly, 1 μL of 10 mg/mL enzyme was incubated with buffer solutions containing 20 μL of 10 mM mannooligosaccharide or 10 μL of 10 mM Man-GlcNAc. The mannooligosaccharides tested were mannotriose (M3), mannotetraose (M4), mannopentaose (M5), and mannohexaose (M6). The reactions were carried out at 40°C for 24 h with samples withdrawn at different time points (0, 1, 3, 6, 9, 12, 24 h), and terminated by heating the samples at 100°C in a water bath for 10 min. After centrifugation at 10,000×g for 10 min, the samples were spotted on silica 60 F254 plates and air-dried. TLC was performed using a mobile solvent containing butanol:propanol:ethanol:water (1:2:2:1). The silica plates were developed by spraying with thymol solution and heating at 120°C for 10 min. The control samples were treated identically to the experimental samples, with the exception of adding *Ca*Man5_18 that had been heat-inactivated at 100 °C for 10 min.

### MALDI-TOF MS analysis of reaction products

Matrix-assisted laser desorption ionization time-of-flight mass spectrometry (MALDI-ToF MS, Applied Biosystems, CA, USA) was used to identify the products resulting from hydrolysis of Man-GlcNAc catalyzed by *Ca*Man5_18. For sample preparation, control samples or reaction product (2.5 μL) were mixed with 10 mM NaCl (6 μL) and 2,5-dihydroxybenzoic acid (DHB) (10 mg/mL, 10 μL) in 50% (v/v) acetonitrile. Then 2 μL of the mixture was spotted onto a stainless steel plate and rapidly dried under vacuum for homogeneous crystallization. MALDI-TOF mass-spectrometry analysis was performed using an accelerating voltage of 20,000 V with a delay time of 200 ns, and operating the instrument under the reflectron mode.

## Results

### Screening of candidate genes for *N*-glycan degradation

To identify a possible *C*. *acnes* degradation pathway for *N*-glycans, the genome of *C*. *acnes* strain 266 (phylogenetic group IA1), was screened for genes coding for carbohydrate-active enzymes (CAZymes) classified in the CAZy database. By applying the operon-prediction method by Price and co-workers [32], we identified an operon corresponding to a possible pathway for cytoplasmic trimming of the Man_3_-GlcNAc_2_ core. The operon contains a GH38 gene (AEE72694) with putative exo-*α*-1,3- or exo-*α*-1,6-mannosidase activity (EC 3.2.1.24), and a GH5 (subfamily 18) gene (AEE72695) coding for a predicted *β*-mannosidase (EC 3.2.1.25). These two enzyme activities are essential for deconstructing the fundamental Man_3_-GlcNAc_2_ core structure, and the absence of a predicted signal peptide suggests that these gene products are located in the cytoplasm. The GH38 and GH5_18 genes cluster together in all *C*. *acnes* strains, beyond which variation occurs in accessory functions. In addition, we note that this gene pair is present in genomes of other bacteria associated with the human microflora, such as *Bifidobacterium breve*, *Bifidobacterium longum*, *Dermabacter vaginalis* and *Arcanobacterium haemolyticum*.

**Fig 2:**
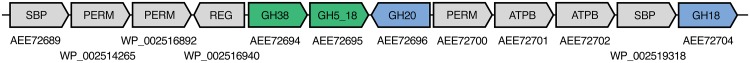
Proposed *C*. *acnes* 266 *N*-glycan-processing locus 1. Gene organization of the proposed *N*-glycan processing locus in the genome of *C*. *acnes* 266. GH genes predicted as mannosidases are colored green, and GH genes with predicted *N*-acetylhexosaminidase activity are blue. Other associated genes are colored light gray and include: predicted sugar ABC-transporter substrate-binding protein (SBP), sugar ABC-transporter permease (PERM), transcriptional-regulator gene (REG), and ABC-transporter ATP-binding protein (ATPB). Accession numbers (GenBank, or RefSeq when GenBank was not available) are shown below each gene.

In the *C*. *acnes* strain 266, the GH38/GH5_18 locus contains two additional GH genes (Fig 2): *(i)* a GH20 (AEE72696) predicted to be an exo-*β*-1,4-*N*-acetylhexoaminidase, which would be able to cleave between the GlcNAc residues in the chitobiose core, and *(ii)* a GH18 (AEE72704) that may act as an endo-*β*-*N*-acetylglucosaminidase (EC 3.2.1.96). However, as shown by comparative genomics [44], the GH20 and GH18 genes are part of a genomic deletion in *C*. *acnes* strains belonging to the phylogenetic groups IB, II and III (S1 Fig), showing that the gene organization observed in strain 266 is not conserved. In addition to the GH genes, the locus contains relevant genes coding for putative sugar ABC transporter permeases and ABC transporter substrate-binding proteins.

**Fig 3:**
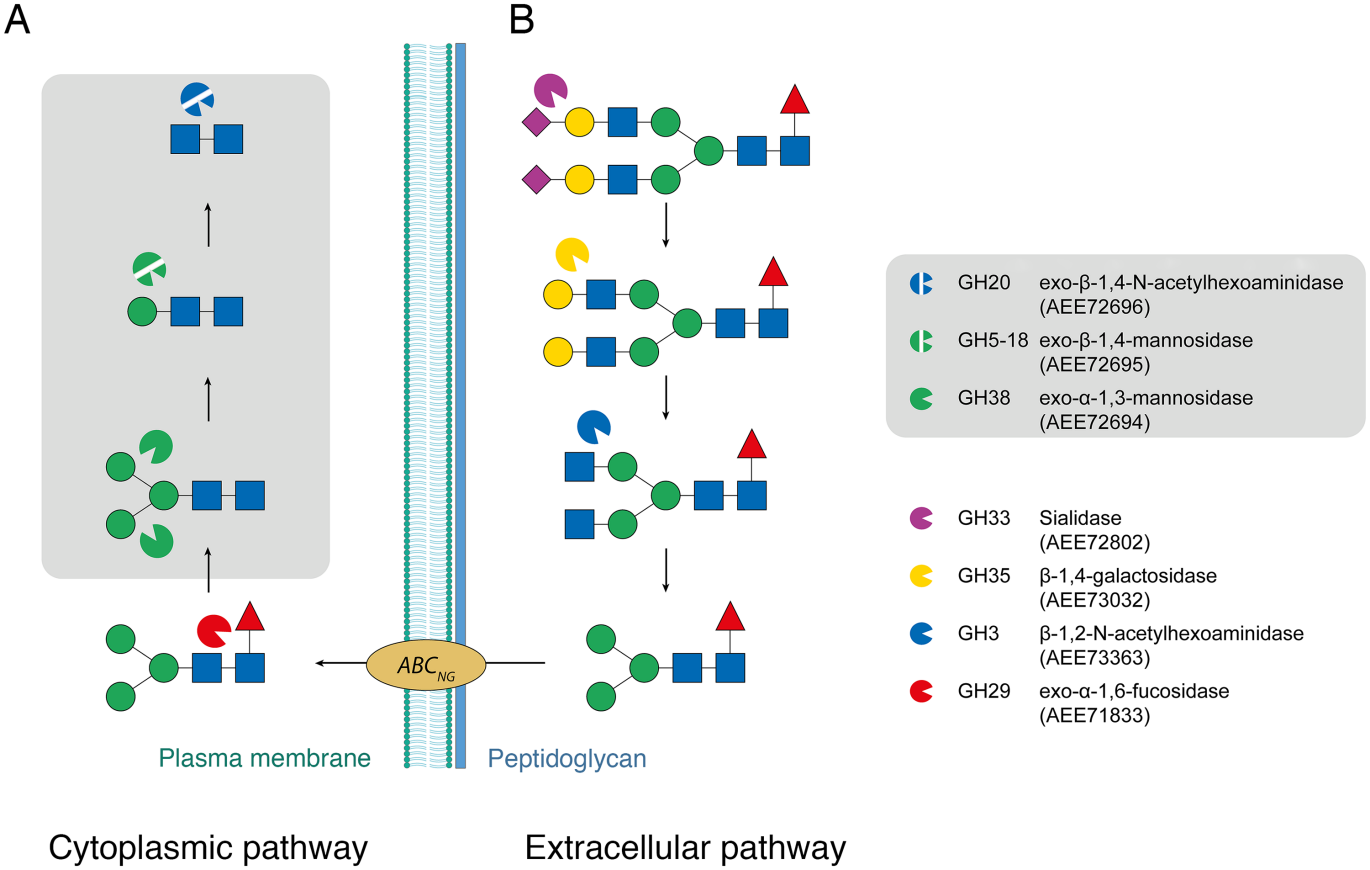
Proposed degradation pathway for host *N*-glycans by *C*. *acnes*. (A) Proposed cytoplasmic pathway involving the enzymes GH38, GH5_18 and GH20, and (B) a hypothetical extracellular pathway. Symbols: blue square: *N*-acetylglucosamine; green circle, mannose; yellow circle, galactose; purple diamond, sialic acid; red triangle, fucose.

Provided that the enzyme activities predicted by CAZy are correct, the locus containing the triad GH38/GH5_18/GH20 would indeed be sufficient to depolymerize the Man_3_-GlcNAc_2_ core into monosaccharide components (Fig 3A). Taken together, these observations strongly suggest that this gene cluster coincides with a carbohydrate-processing locus (Fig 2). Separate from this locus, we also identified a GH29 gene (AEE71833) that lacks secretion signal, and is predicted to code for an exo-*α*-1,6-fucosidase, an activity relevant for removal of fucose from *N*-glycans that are fucosylated at the chitobiose core.

Identification of candidate genes coding for secreted enzymes that could participate in the initial trimming of the *N*-glycan on the extracellular side is more speculative, but three GH genes that contain signal-peptide sequences deserve particular attention, namely genes coding for a GH33 (AEE72802), GH3 (AEE73363), and a GH35 (AEE73032) (Fig 3B). The GH33 gene (AEE72802) displays 59% sequence identity to the *nedA* gene from *Micromonospora viridifaciens* (UniProt Q02834), which codes for the enzyme NanH that catalyzes removal of terminal sialic acid in glycoconjugates (sialidase; EC 3.2.1.18) [45]. The *C*. *acnes* GH33-gene sequence corresponds mainly to the catalytic sialidase domain that folds as a six-bladed β-propeller [46], and lacks the accessory domains present in *M*. *viridifaciens* NanH (*i*.*e*., a linking NPCBM domain and a sugar-binding F5/8-type C domain). Importantly, all catalytically important amino acids, and the five bacterial neuraminidase repeats (BNR repeats; Asp-box motifs [47]) are present. The GH33 sequence in *C*. *acnes* 266 displays a frame shift at the C-terminus, however, the corresponding genes in high-quality genomes of the closely related *C*. *acnes* strains PA_12_1_L1 and PA_12_1_R1 are present as full-length versions suggesting that the 266 sequence is incorrect due to gene sequencing or assembly problems. Still, even after correcting for this error, the *C*. *acnes* GH33 sequence beyond the catalytic domain is different from that of NanH.

The *C*. *acnes* 266 GH3 gene (AEE73363) is predicted to code for a *β*-1,2-*N*-acetyl-hexosaminidase (EC 3.2.1.52), which would be a suitable candidate for cleavage of GlcNAc from an *N*-glycan. The GH3 gene shows 36% sequence identity to NagZ (YbbD) from *Bacillus subtilis* strain 168 (UniProt P40406), and also contains the required catalytic Asp-His dyad [48]. In line with our hypothesis, this GH3 enzyme has been shown to be part of the *C*. *acnes* secretome [26].

The third required activity in the extracellular path of complex and hybrid *N*-glycan degradation by *C*. *acnes* is the exo-*β*-1,4-galactosidase (EC 3.2.1.23) to remove *β*-1,4-linked galactose. The only relevant gene candidate with a signal-peptide sequence (AEE73032) belongs to family GH35. The gene is annotated as a fragment in the CAZy database, but closer inspection of the sequence reveals that it is 32% identical to the eukaryotic N-terminal *β*-galactosidase (*β*-Gal) TIM-barrel domain of Penicillium sp. (PDB code 1XC6 [49], residues 41–265), with the active site and most galactose-binding residues conserved. The GH35 gene is absent in a few *C*. *acnes* strains, but when present, including in high-quality genomes [29], it always occurs in this short version. Hence, the observed shorter GH35 sequence is not likely due to a sequencing error. We find several examples in various *C*. *acnes* genomes of a gene combination with a short GH35 module and a GH20 module. There is also considerable variation in the genomic location of the GH35 gene across *C*. *acnes* strains, and occasionally, a second short GH35 gene is encountered, sometimes with one or two GH85 genes that encode fragments or full-length proteins. We thus hypothesize that *C*. *acnes* strains employ a minimal structural *β*-Gal framework for the purpose of trimming off galactose units from *N*-glycans. It should be noted that while all three proposed extracellular enzymes in *C*. *acnes* 266 contain a secretion signal, neither has a typical C-terminal cell-wall anchoring LPxTG motif. However, this motif does not seem to be consistently present in secreted glycan-processing enzymes. For instance, the GH35 *β*-galactosidase BgaC from *Streptococcus pneumoniae* has been shown to localize to the cell surface although the protein lacks a typical signal peptide and LPxTG sequence motif [50], which highlights differences in sorting mechanisms among Gram-positive bacteria.

### Phylogenetic analysis of the *Ca*Man5_18 sequence

The AEE72695 gene coding for the GH5_18 enzyme (408 amino acids) was predicted to be a *β*-mannosidase based on the GH5-subfamily classification in CAZy [51]. A BLAST search with the AEE72695 sequence against the Protein Data Bank did not return any sequence hits for proteins with known three-dimensional structure. We speculated that this gene product might be responsible for cleaving off Man from the Man-GlcNAc_2_ core, since this bond is the only mannose-linking glycosidic bond in human *N*-glycans in *β* configuration. In fact, the *β*-1,4 bond in Man-GlcNAc is the only conceivable substrate for a *β*-mannosidase involved in host *N*-glycan metabolism.

Although subfamily GH5_18 includes 178 actinobacterial sequences (as of May 2018), no biochemically confirmed substrate specificity has been reported for any of the enzymes [51]. There is one exo-mannosidase characterized that belongs to the most closely related subfamily GH5_19 (EC 3.2.1.25) [52], as well as other mannan-active enzymes that are members of subfamilies GH5_7, GH5_10, GH5_17 and GH5_31. Together with GH5_18 and GH5_19, these make up one of the three major clades of the GH5 family, and it has been proposed that members of these subfamilies are likely to act on mannose-containing carbohydrates [51].

Subfamily GH5_18 is dominated by sequences from three genera: *Cutibacterium* (21 sequences), *Bifidobacterium* (51 sequences) and *Streptomyces* (51 sequences). All available genomes of *C*. *acnes* strains present in the CAZy database contain a GH5_18 gene. A phylogenetic tree of GH5_18 sequences shows that *Ca*Man5_18 falls in a clade with other enzymes from various *C*. *acnes* strains, as well as sequences from *C*. *avidum* and *Cutibacterium* sp. oral taxon 193 (S2 Fig). In addition, this clade contains proteins from *Acidipropionibacterium acidipropionici* ATCC 4875 and *Tessaracoccus bendigoensis* DSM 12906. The latter species belong to the same family, *Propionibacteriaceae*, as the *Cutibacterium* genus. There are minor sequence variations between different *C*. *acnes* phylotypes (S3 Fig), where the largest discrepancy is observed at the C-terminus in different strains. The C-terminus in our *Ca*Man5_18 structure (residues 396–408) is flexible and lacks interpretable electron density. This region is located far away from the active site and dimer interface, which means that it does not play a direct role in substrate hydrolysis or dimer formation in either our *Ca*Man5_18 (IA strain), or in the homologous enzymes of type IB, II and III strains. The most consistent difference among sequences of the *Cutibacterium* clade and other GH5_18 proteins is a short insert of four residues (^149^PGHH^152^, *Ca*Man5_18 numbering) downstream of the catalytic residue Glu140 (S4 Fig).

### Overall structure and active site of *Ca*Man5_18

**Fig 4:**
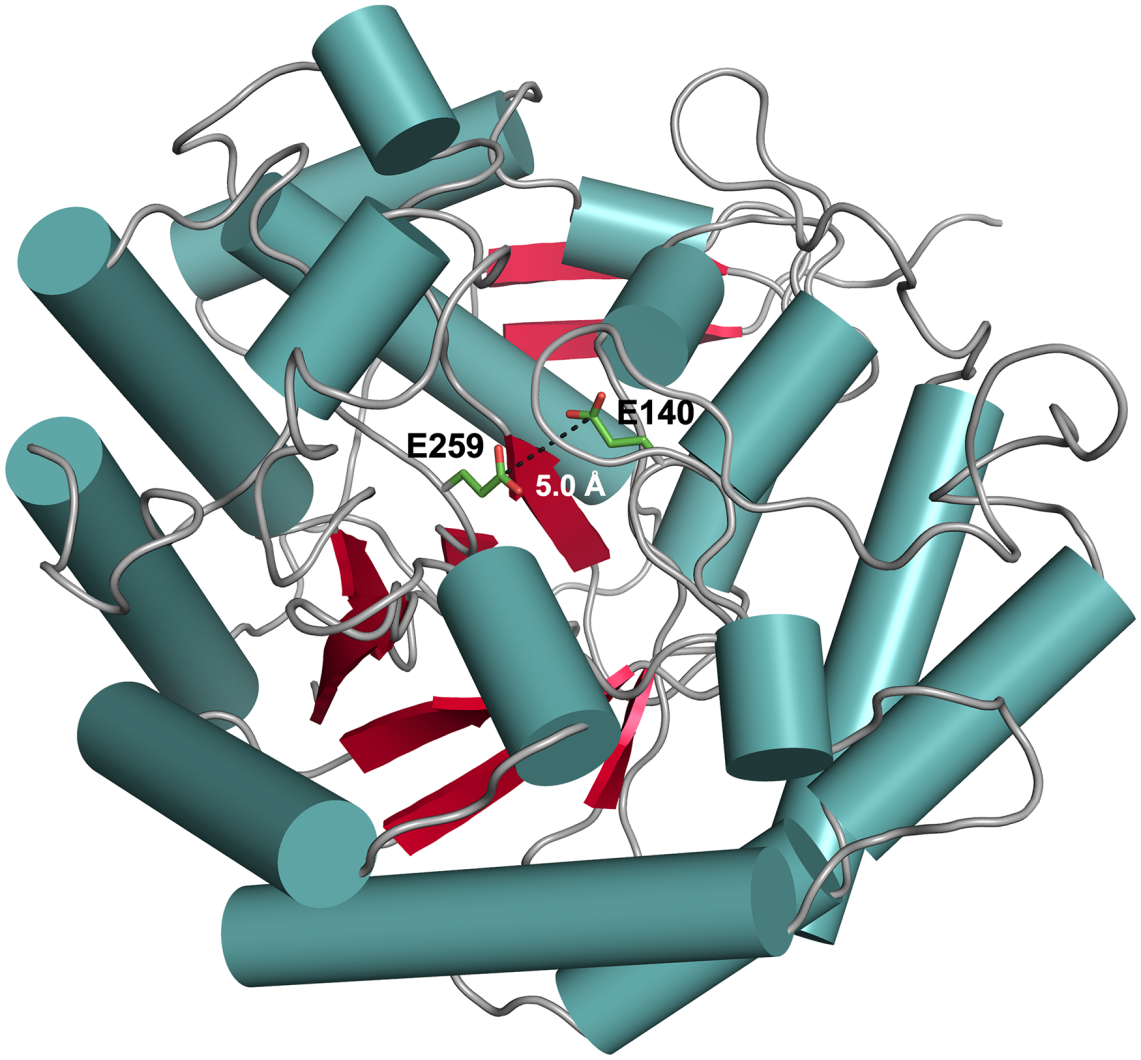
Ribbon representation of the *Ca*Man5_18 subunit structure. The subunit structure of *Ca*Man5_18 features a (β/α)_8_ TIM-barrel fold typical for members of the GH5 family. The catalytic acid/base Glu140 and the nucleophile Glu259 are represented as stick models. Secondary-structure elements are represented as: α-helices, blue cylinders; β-sheets, red arrows; and loops, gray coils.

The crystal structure of *Ca*Man5_18 was determined and refined in the unliganded state at a final resolution of 1.8 Å (Table 1). The enzyme was co-crystallized with mannose, however, no clear density for the sugar could be identified. The overall structure displays the, for GH5 enzymes typical, (β/α)_8_ TIM-barrel fold. Members of the GH5 family share two conserved glutamate residues at the active site, which are approximately 5 Å apart and buried some 12 Å below the protein surface (Fig 4). Using the DALI server (http://ekhidna2.biocenter.helsinki.fi/dali/) to identify structurally similar proteins, the top 20 three-dimensional structures show low sequence identities to *Ca*Man5_18 in the range 10–19%, and high root-mean-square deviation (r.m.s.d) values for atomic positions in the range 2.9–3.3 Å. This confirms the initial, unsuccessful attempts to identify structural homologs using BLAST.

**Fig 5:**
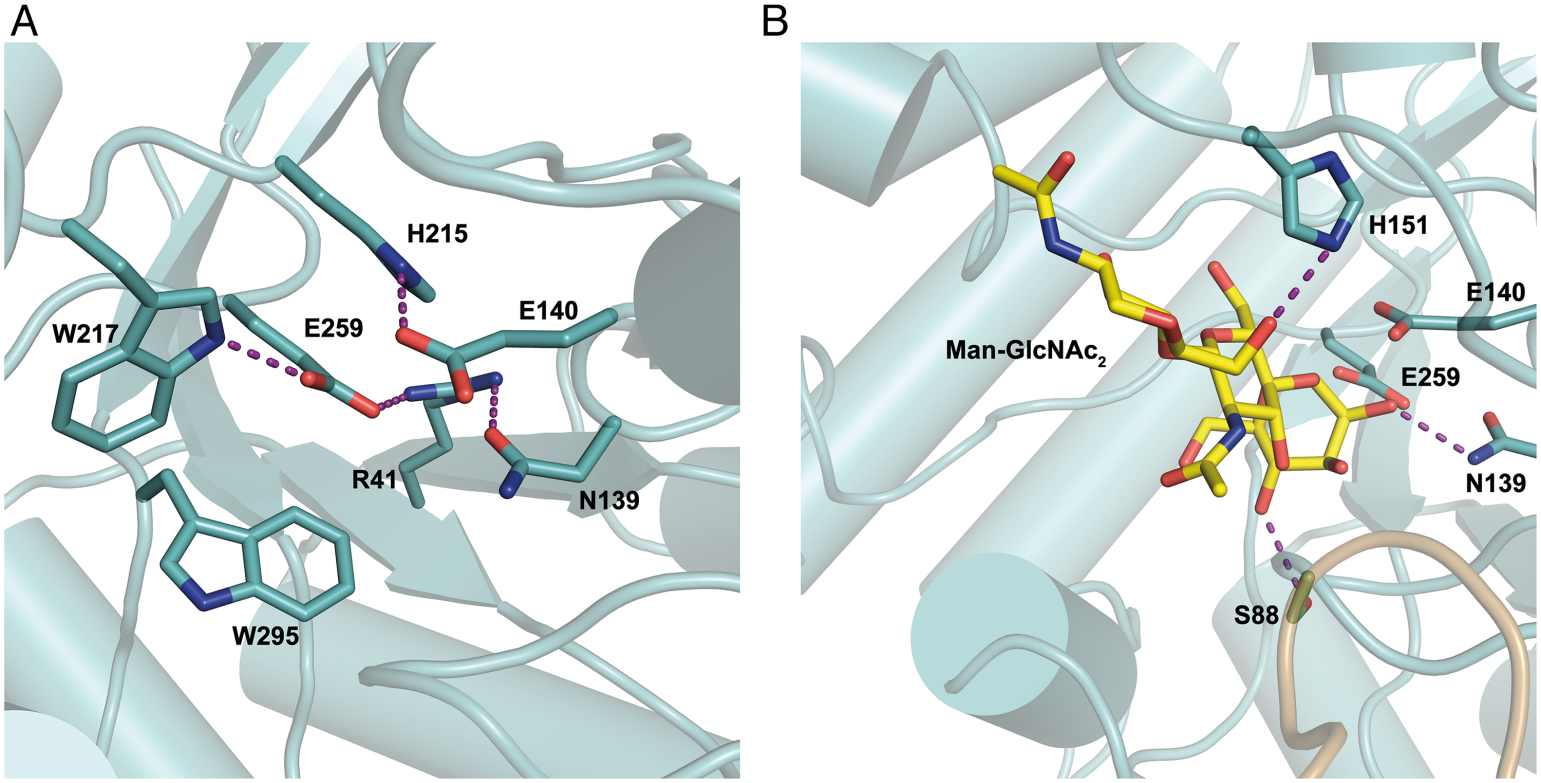
Close up of the active site in *Ca*Man5_18. (A) Interactions made by the catalytic residues Glu140 and Glu259 with Arg41, Asn139, His215, Trp217 and Trp295. (B) Hypothetical binding of Man-GlcNAc_2_ and possible interactions with Ser88, Asn139 and His151. Purple dashed lines highlight interactions.

With the aim to capture a crystal complex of wild-type *Ca*Man5_18 with the Man-GlcNAc substrate, we produced the E140Q/E259Q mutant where the catalytic glutamates had been replaced by their isosteric amide counterparts. Unfortunately however, the mutant did not crystallize. Furthermore, we were unsuccessful in capturing a complex of *Ca*Man5_18 with mannose, probably because a disordered MOPS molecule is present in the active site of the crystal structure. Although the experimental data do not show bound ligand in the active site, we can derive from close homologs that Glu140 is acting as the acid/base catalyst and Glu259 as a nucleophile (S4 Fig). The conformation of Glu140 is supported by interactions with His215, and the neighboring Asn139 is kept in position through a possible hydrogen bond with Arg41. The orientation of the catalytic nucleophile Glu259 can be stabilized through interactions with Arg41 and Trp217 (Fig 5A).

To analyze possible substrate-protein interaction the proposed substrate, Man-GlcNAc_2_ (see product analysis below), was docked manually in the active site of *Ca*Man5_18 (Fig 5B). Briefly, the Man-GlcNAc_2_ compound was generated manually by using the coordinates for the corresponding sugar units in the crystal structure of a glycosylated IgG Fc (PDB code 5DI8, chain A [53]). Furthermore, the mannose in subsite –1 was modified to adopt the conformation observed for the corresponding mannose in the crystal structures of *Rhizomucor miehei* Man5 (PDB codes 4NRR and 4NRS [54]). Torsion angles of the GlcNAc units of the chitobiose core were adjusted manually in COOT [41] to avoid unfavorable contacts with proteins atoms in subsites +1 and +2. Based on this hypothetical binding mode, the side chains of Ser88 and Asn139 are predicted to interact with the 4-hydroxyl and 2-hydroxyl oxygen of a mannose residue in subsite –1, respectively. Additionally, His151 is suitably placed to interact with the terminal GlcNAc unit (Fig 5B). The C-terminus (394–408) of the experimental structure does not show interpretable electron density, probably due to flexibility, and was therefore not modeled.

### Analysis of oligomeric state

**Fig 6:**
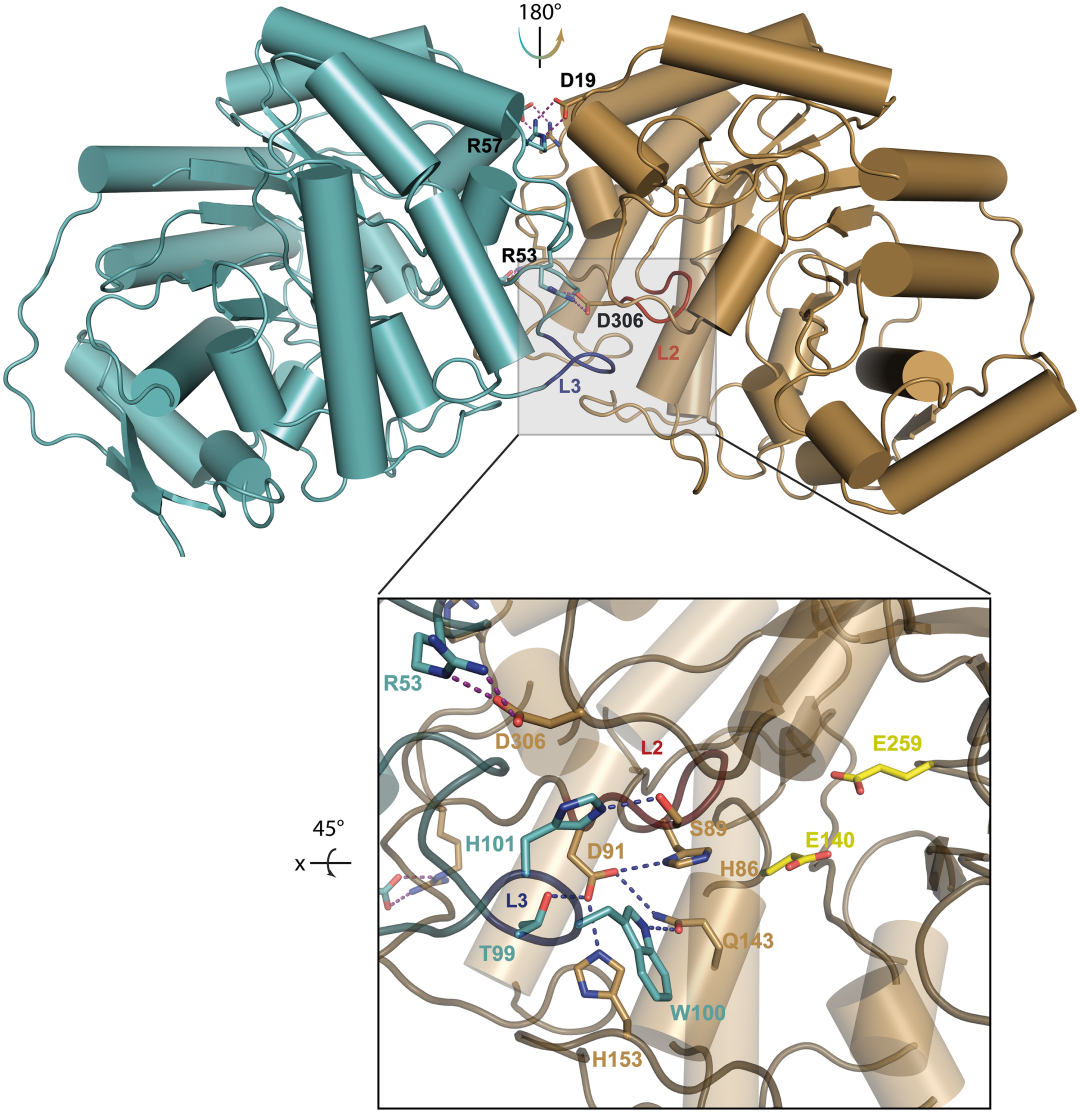
Dimerization of *Ca*Man5_18. The dimer interface between subunit A (cyan) and subunit B (brown) with the side chains highlighted that stabilize the dimeric state through intersubunit salt links (Asp19-Arg57 and Arg53-Asp306). Loops L2 (red; residues 89–92) and L3 (blue; residues 92–102) participate in dimer formation, as well as in forming the blockage of the active site required for exo-mode activity. Inset: Interactions formed by loops L2 and L3 in the active site. The steric blockage is mainly provided by L2, supported by L3 and L5 (residues 299–314). The catalytic residues Glu140 and Glu259 are colored yellow.

To determine the oligomeric state of the *Ca*Man5_18 *in vitro*, we applied several techniques: *(i)* analysis of crystal contacts between *Ca*Man5_18 monomers, *(ii)* SEC analysis, and *(iii)* inspection of the protein unfolding profile as a function of temperature. The enzyme is present as a two-fold symmetrical homodimer in the crystal (Fig 6), featuring an extensive dimer interface generated by five loop regions: L1 (residues 46–57), L2 (residues 89–92), L3 (residues 98–102), L4 (residues 152–156), and L5 (residues 299–314), where especially L2 and L3 shape and stabilize the active site (Fig 6, inset). Ion links can be established between Asp19 and Arg57 as well as between Arg53 and Asp306 from both monomers across the two-fold-symmetrical dimer interface.

Analysis of the dimer interface using PISA (Proteins, Interfaces, Structures and Assemblies; http://www.ebi.ac.uk/pdbe/pisa/) [55], supports a stable dimeric state with a buried surface area of 3270 Å^2^ and a complex-formation significance score of 1.0. An estimated solvation free energy gain (Δ*G*^int^), *i*.*e*., the gain in solvation free energy upon dimer formation calculated as the difference in solvation energy between free monomers and the dimer state, of –20.8 kcal/mol; and a free energy of assembly dissociation (Δ*G*^diss^), *i*.*e*., the free-energy difference between dissociated and associated states, of 21.3 kcal/mol.

In agreement with a dimeric protein, the unfolding profiles observed for *Ca*Man5_18 during thermofluor denaturation experiments are biphasic, as exemplified by two unfolding transitions at pH 7.5 corresponding to *T*_m_ values of 48.8°C and 58.0°C (Parts A and B in S5 Fig). Further evidence for a natural dimer in solution was provided by the result from SEC analysis. Whereas the molecular weight of monomeric *Ca*Man5_18 is 45.7 kDa, the protein elutes as a 98-kDa species on a Superdex 200 column (Part C in S5 Fig).

### Substrate specificity

Initially, the activity of *Ca*Man5_18 was investigated by screening different *p*NP-glycosides as substrates. Of the *p*NP sugars, only *p*NP-*β*Man served as substrate for *Ca*Man5_18 with a specific activity of 2.6 U (Table 2). Possible endo activity was tested on relevant complex substrates, *e*.*g*., mannan and glucomannan, however, no hydrolysis was observed, which indicated that *Ca*Man5_18 is an exo-*β*-mannosidase.

**Table 2:** Substrate screening.

Substrate	Specific activity (U)
*p*NP-*β* Man	2.6 ± 0.13
*p*NP-*β* Xyl	ND
*p*NP-*β* Fuc	ND
*p*NP-*α* Fuc	ND
*p*NP-*β* Gal	ND
*p*NP-*β* Glc	ND
*p*NP-*β* cellobiose	ND
Mannan	ND
Glucomannan	ND
Xylan	ND
Barley *β*-glucan	ND
CMC	ND
Cellobiose	ND

ND, not detected.

Furthermore, the kinetic parameters (*V*_max_, *K*_M_, *k*_cat_ and *k*_cat_*/K*_M_) were determined for *Ca*Man5_18 with *p*NP-*β*Man as substrate under the conditions of pH_opt_ and *T*_opt_ for *in-vitro* activity (see below). For *p*NP-*β*Man, a Michaelis constant, *K*_M_, of 10.4 mM was obtained, and a turnover number, *k*_cat_, of 7.76 s^-1^, corresponding to a catalytic efficiency of 0.746 s^-1^ mM^-1^. Since the only purpose of the mutant was to attempt trapping of a protein-substrate co-crystal complex, no further biochemical or kinetic characterization was performed.

### Reaction pH, kinetic stability and thermal stability

**Fig 7:**
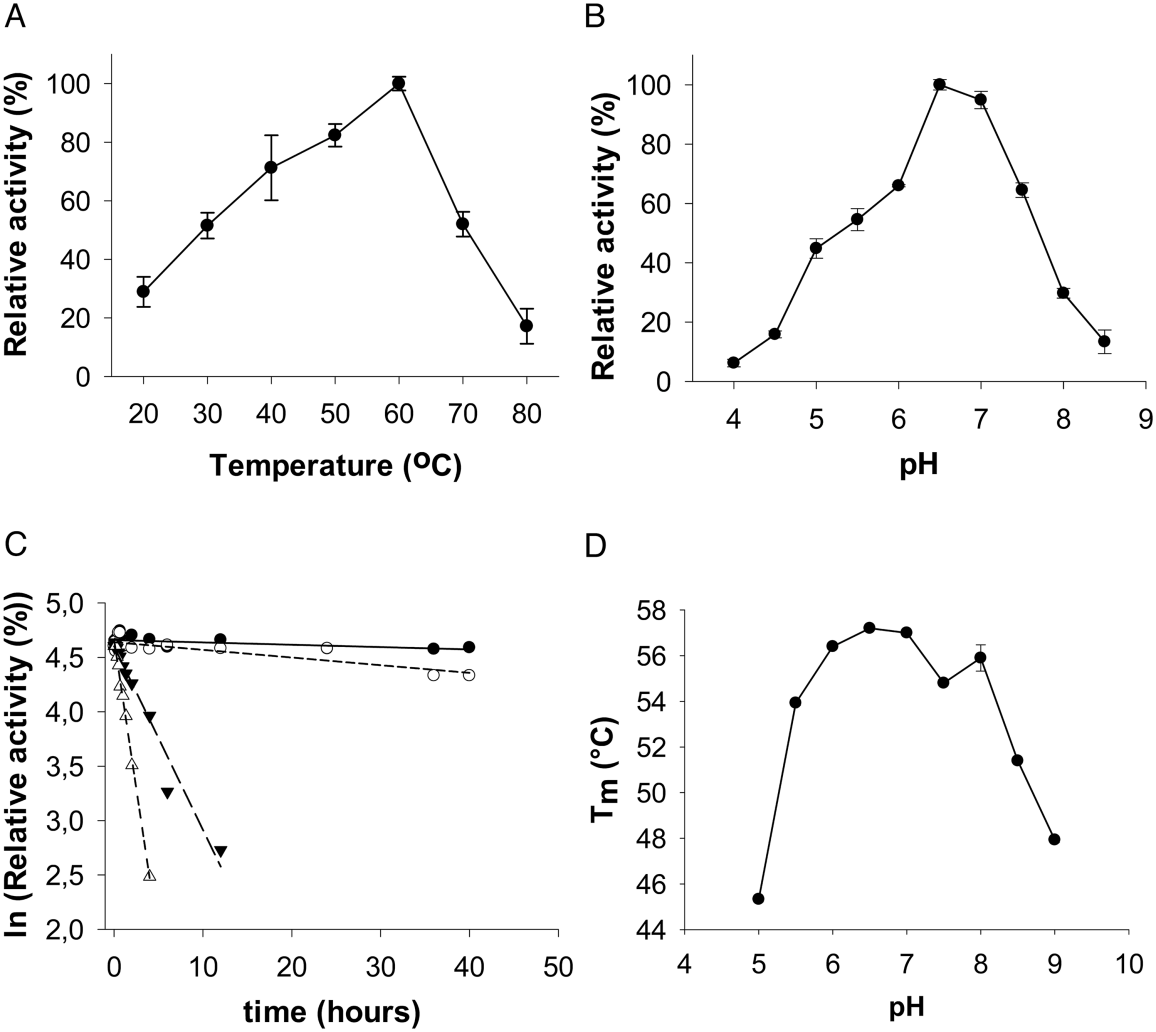
*Ca*Man5_18 activity on *p*NP-*β*Man. Dependency of *Ca*Man5_18 activity with *p*NP-*β*Man as substrate on (A) temperature, and on (B) pH. (C) Kinetic stability of *Ca*Man5_18 hydrolysis of *p*NP-*β*Man. Symbols: filled circles, 40°C; empty circles, 50°C; filled triangles, 60°C; empty triangles, 70°C. (D) ThermoFluor analysis of thermal stability to unfolding as a function of pH.

Optimum temperature and pH for *p*NP-*β*Man hydrolysis by *Ca*Man5_18 was determined to be 60°C (Fig 7A), and 6.5 (Fig 7B), respectively. The activity decreases by 50% at temperatures above 70°C, and declines rapidly below 60% at pH values near the narrow pH_opt_. Analysis of the kinetic stability shows that the activity remains stable at lower temperatures, 40°C and 50°C, over an extended period of time with half-life time values of 322 h and 98 h, respectively. At higher temperatures, 60°C and 70°C, the kinetic stability is reduced, as evidenced by half-life time values of 4 h and 1.2 h, respectively (Fig 7C). ThermoFluor analysis of the thermal stability to unfolding as a function of pH showed that the enzyme’s structural integrity remains intact within the pH range 6.5 to 8.0, and that *T*_m_ decreases rapidly at higher pH values (Fig 7D).

### Product analysis

**Fig 8:**
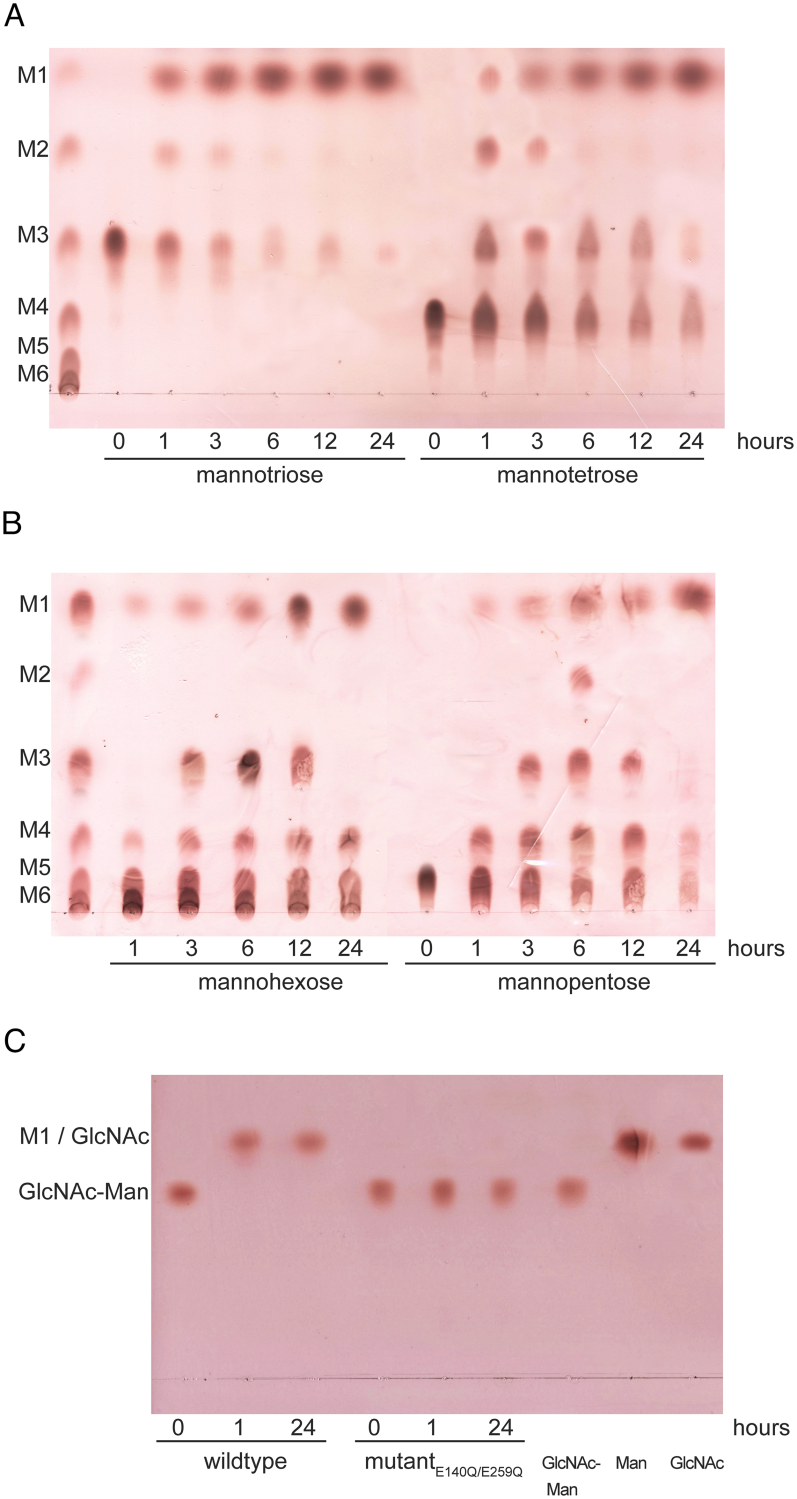
TLC analysis of product formation. Separation of the hydrolysis products from time-dependent hydrolysis of mannooligosaccharides and Man-GlcNAc by wild-type *Ca*Man5_18. (A) M3 and M4; (B) M6 and M5 with the mannooligosaccharide standards M1-M6 at the left; (C) Man-GlcNAc hydrolysis by *Ca*Man5_18 wild type and the E140Q/E259Q double mutant.

Released monosaccharides and oligosaccharides upon enzyme treatment were identified using TLC and MALDI-TOF mass spectrometry. As stated above, the initial substrate screening of *p*NP sugars indicated hydrolytic activity only for *β*-1,4-linked mannose. Time-dependent TLC analyses of hydrolysis products from *β*-linked mannooligosaccharides of degree of polymerization (DP) of 3 to 6 revealed that M1 (mannose) is accumulating slowly with time for all substrates tested (Fig 8).

For the mannotriose (M3) as substrate (Fig 8A), the products M1 and M2 appear early, after 1 h. Accumulation of M2 occurs at 1–3 h, and is depleted at 3–6 h. After 24 h, most M3 substrate has been converted to M1. For mannotetraose (M4) as substrate (Fig 8A), the products M1, M2 and M4 appear after 1 h. As for the M3 substrate, accumulation of M2 takes place at 1–3 h, and is depleted at 3–6 h. After 24 h, most of the M4 substrate has been converted to M1 and some residual M3, but even at 24 h, residual M4 substrate remains.

For mannopentaose (M5) as substrate (Fig 8B), small amounts of M1 and some M4, but no M2 or M3, are formed early, after 1 h. Concomitantly with conversion of M5 substrate, we observe an accumulation of M1, M3 and M4 at 3–12 h, and small amounts of M2 appearing after 6 h. After 24 h, the product mixture is dominated by M1 and residual M4 and some remaining M5 substrate. For mannohexaose (M6) as substrate (Fig 8B), small amounts of M1, M4 and some M5, but no M2 or M3, are formed early, after 1 h. Concomitantly with conversion of M6 substrate, there is an accumulation of M1, M3, M4 and M5 at 3–12 h, but no M2. After 24 h, the product mixture is dominated by M1 and residual M4, M5 and considerable amounts of M6 substrate. The result of the product analysis of *β*-1,4-linked mannooligosaccharides is consistent with *Ca*Man5_18 being an exo-*β*-1,4-mannosidase.

**Fig 9:**
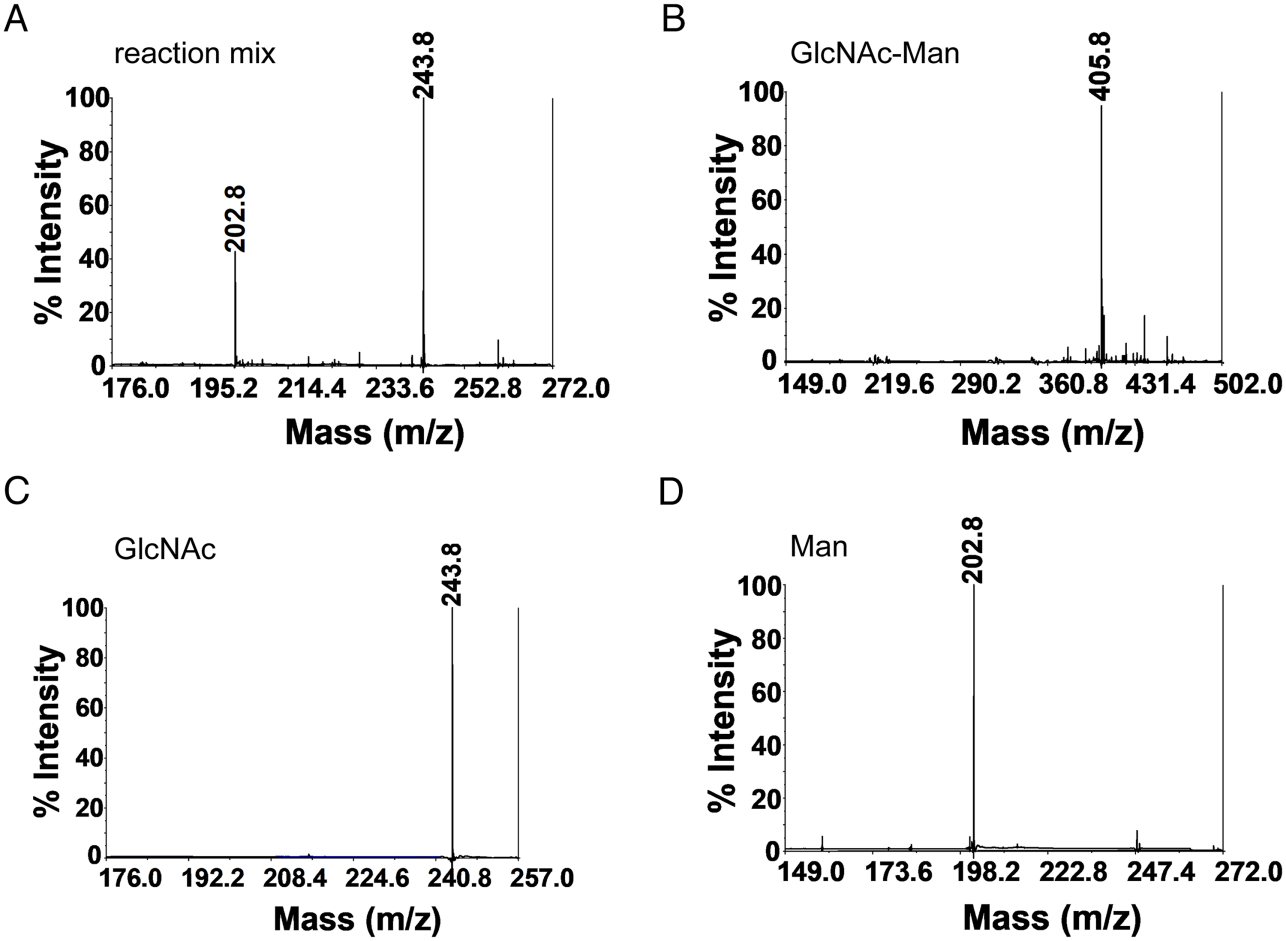
MALDI—TOF-MS analysis of Man-GlcNAc hydrolysis. (A) Enzymatic reaction products using Man-GlcNAc as substrate after 1 h of incubation. Controls: (B) Man-GlcNAc; (C) GlcNAc; and (D) Man.

For the Man-GlcNAc substrate that corresponds to the relevant substrate in an *N*-glycan, full conversion of the substrate to Man and GlcNAc was achieved at 1 h, *i*.*e*., the earliest time point tested, and as expected, the *Ca*Man5_18 mutant E140Q/E259Q was unable to hydrolyze the substrate (Fig 8C). Since the products Man and GlcNAc co-migrated on TLC, MALDI-TOF mass spectrometry successfully confirmed the product identities (Fig 9).

## Discussion

In this work, we propose gene candidates likely to code for enzyme activities involved in a cytoplasmic pathway for degradation of the Man_3_-GlcNAc_2_ *N*-glycan core structure (Fig 3A, Table 3). The gene coding for *Ca*Man5_18 clusters with two genes: AEE72694 (predicted GH38; exo-*α*-1,3- or exo-*α*-1,6-mannosidase; EC 3.2.1.24) and AEE72696 (predicted GH20; exo-*β*-1,4-*N*-acetylhexoaminidase; EC 3.2.1.52), located immediately upstream and downstream of the *Ca*Man5_18 gene, respectively (Fig 2). We find it legitimate to propose that these three genes, together with nearby genes annotated as sugar transporters and sugar-binding proteins, are part of a distinct locus with relevance to an *N*-glycan degradation pathway (Figs 2 and 3A). Indeed this enzyme triad would be able to completely convert the Man_3_-GlcNAc_2_ core to monosaccharides on the cytoplasmic side.

**Table 3:** Genes implied in *N*-glycan degradation (NGD) by *C*. *acnes* strain 266.

Gene accession code	CAZy family	Secretion signal	EC number	Predicted activity	NGD locus	Pathway	Representative homolog with known 3-D structure (sequence identity) [citation]
AEE72694	GH38	No	3.2.1.24	exo-*α*-1,3- mannosidase, exo-*α*-1,6-mannosidase	1	Cytoplasmic	Ams1, UniProt P22855, PDB 5JM0 (30.1%) [56] SpGH38, UniProt Q99YP5, PDB 2WYH (22.5%) [57]
AEE72695	GH5_18	No	3.2.1.25	exo-*β*-1,4-mannosidase (confirmed)	1	Cytoplasmic	This work, 6GVB; no available structurally characterized homologs with sequence identity > 19%
AEE72696	GH20	No	3.2.1.52	exo-*β*-1,4-*N*-acetylhexosaminidase	1	Cytoplasmic	SP_2141, UniProt A0A0H2US73, PDB 5AC4 (20.0%) [58]
AEE72704	GH18	No	3.2.1.96	endo-*β*-*N*-acetylglucosaminidase	1	Cytoplasmic	endoT, UniProt C4RA89, PDB 4AC1 (41.7%) [59]
AEE71833	GH29	No	3.2.1.51	*α*-L-fucosidase	–	Cytoplasmic	TM_0306, UniProt Q9WYE2, PDB 1HL8 (27.1%) [60] BT2970, UniProt Q8A3I4, PDB 2WVS (24.9%) [61]
AEE71274	GH4	No	3.2.1.86	6-phospho-glucosidase	2	Cytoplasmic	Gan4C, UniProt W8R9V4, PDB 5C3M(31.6%) [N/A]
AEE71275	GH38	No	3.2.1.24	exo-*α*-1,3- mannosidase, exo-*α*-1,6-mannosidase	2	Cytoplasmic	Ams1, UniProt P22855, PDB 5JM0 (30.0%) [56] SpGH38, UniProt Q99YP5, PDB 2WYH (23.5%) [57]
AEE71278	GH125	No	3.2.1.–	exo-*α*-1,6-mannosidase	2	Cytoplasmic	CPE0426/CpGH125, UniProt Q8XNB2, PDB 3QT3 (44.5%) [62] SP_2144/SpGH125, UniProt A0A0H2URZ6, PDB 3QPF (42.6%) [62]
AEE71279	GH20	No	3.2.1.52	exo-*β*-1,4-*N*-acetylhexosaminidase	2	Cytoplasmic	nahA, UniProt A1RBZ5, PDB 3RCN (36.5%) [N/A]
AEE72802 frameshift*	GH33	Yes	3.2.1.18	sialidase	–	Extracellular	nedA/NanH, UniProt Q02834, 1EUS (59.0%) [45]
AEE73363	GH3	Yes	3.2.1.52	*β*-1,2-*N*-acetylhexosaminidase	–	Extracellular	nagZ/YbbD, UniProt P40406, 3BMX (34.7%) [48]
AEE73032	GH35	Yes	3.2.1.23	*β*-1,4-galactosidase	–	Extracellular	N-terminal *β*-galactosidase (*β*-Gal) TIM-barrel domain, UniProt Q700S9, 1XC6 residues 41–265 (34.4%) [49]

*) Full-length representative: ALT42513, *C*. *acnes* strain PA_12_1_L1

A similar pathway appears to exist in the human pathogen *Streptococcus pyogenes* where the exo-*α*-mannosidase *Sp*GH38 hydrolyzes mannose *α*-1,3 and *α*-1,6 linkages in host *N*-glycans [57]. However, in *Streptococcus pneumoniae*, the corresponding GH38 is predicted to have exo-*α*-1,3-mannosidase activity, while a GH125 would provide the complementary exo-*α*-1,6-mannosidase activity [21]. Interestingly, we have identified another *C*. *acnes* locus that contains the GH38/GH125 gene pair in combination with genes coding for GH20 and GH4 members (S6 Fig). This pair is conserved in all *C*. *acnes* types except for the type-III strains. While this may indicate that *C*. *acnes* uses alternative pathways for hydrolysis of the *N*-glycan core, this latter locus lacks a candidate gene for the *β*-mannosidase activity.

As a first step towards experimentally confirming our proposed cytoplasmic *N*-glycan-degradation pathway, we chose to characterize the GH5_18 gene product that was predicted to code for a putative *β*-mannosidase. The rationale for this choice was that, unlike the other proposed enzyme activities, GH5 enzymes have not previously been linked to *N*-glycan degradation. The amino-acid sequences of exo-acting *β*-mannosidases (EC 3.2.1.25) are found in any of the three GH families 1, 2 and 5 [30], which belong to the GH-A clan that features the (β/α)_8_ TIM-barrel fold. These enzymes follow a retaining reaction mechanism according to the classical Koshland double-displacement mechanism [63] where two conserved glutamate residues act as acid/base catalyst and nucleophile [64]. Of the 31 *β*-mannosidase sequences that are currently classified in the CAZy database, eight belong to GH1, 21 to GH2, and only two are GH5 members, namely, the exo-*β*-mannosidase *Cm*Man5A from *Cellvibrio mixtus* (UniProt Q6QT42; PDB code 1UUQ [65]) and the exo-*β*-mannosidase *Tth*Man5 from *Pseudothermotoga (Thermotoga) thermarum* (UniProt F7YX66) [52]. While the majority of GH5 enzymes are endo-acting, exo-acting enzymes dominate the GH1 and GH2 families.

The proposed model of *N*-glycan metabolism in *S*. *pneumoniae* lacks a candidate for cleaving the *β*-1,4 linkage in Man-GlcNAc, and in the *N*-glycan-processing pathways of *Xanthomonas campestris* [66] and the human colonic bacterium *Bacteroides thetaiotaomicron* this *β*-mannosidase activity is provided by a GH2 enzyme [67]. In *B*. *thetaiotaomicron*, the gene coding for the *β*-mannosidase is located in a polysaccharide-utilization locus (PUL), which also contains three GH20 genes, a GH2 gene and a putative GH33 sialidase gene. A related GH2 enzyme in *Bacteroides fragilis* predicted to degrade the *N*-glycan core displays *β*-mannosidase activity, although activity on Man-GlcNAc or Man-GlcNAc_2_ has not been demonstrated [68].

Of all aryl-glycosides and polysaccharide substrates tested, *Ca*Man5_18 showed activity only for *p*NP-*β*Man (Table 4). Activity on aryl-mannosides such as *p*NP-*β*Man is the generally accepted way to distinguish exo-acting mannosidases from endo-acting mannanases [65]. The exo-*β*-mannosidases *Bt*Man2A [67] and *Tth*Man5 [52] perform well with *p*NP-*β*Man as substrate whereas the exo-*β-*mannosidase *Cm*Man5A does not (Table 4). Similar to *Cm*Man5A, *Ca*Man5_18 displays low but convincing activity on *p*NP-*β*Man. However, product analysis by TLC reveals a product pattern consistent with an exo-mannosidase (Fig 8). Although *Ca*Man5_18 can accept mannooligosaccharides (DP 3–6) as substrates, and given enough time, convert most of these to free mannose, the efficiency declines as a function of increasing DP (Fig 8). Furthermore, using M5 and M6 as substrates, neither M2 nor M3 is generated early, *i*.*e*., after 1 h (Fig 8B), which is supportive of an exo-mode activity on *β*-1,4-linked mannooligosaccharides.

**Table 4:** Kinetic parameters for *Ca*Man5_18 and related enzymes on *p*NP-*β*Man.

	*K*_M_ (mM)	*k*_cat_ (s^-1^)	*k*_cat_ / *K*_M_ (s^-1^ mM^-1^)	Reference
*Ca*Man5_18	10.4	7.8	0.75	This work
*Cm*Man5A	1.6	0.033	0.021	[65]
*Tth*Man5	4.4	1924	441	[52]
*Bt*Man2A	0.19	128	674	[67]

In contrast to the mannooligosaccharides, wild-type *Ca*Man5_18 hydrolyzes Man-GlcNAc efficiently to mannose and GlcNAc in 1 h (Fig 8C), while no reaction products were observed when incubating the double mutant E140Q/E259Q with this substrate. This confirms the importance of the proposed catalytic amino acids, as well as the relevance of Man-GlcNAc as substrate. Since both hydrolysis products, Man and GlcNAc, are co-migrating on TLC, further analysis using MALDI-TOF mass spectrometry was needed to confirm the product identities. The results from MALDI-TOF mass spectrometry showed clear separation of Man and GlcNAc, which provided positive confirmation of the hydrolysis products (Fig 9).

Several observations based on the crystal structure also support that Man-GlcNAc_2_ is a more preferred substrate for *Ca*Man5_18 compared with longer mannose-containing substrates: *(i)* loop 2 (chain A, residues 89–92), loop 3 (chain B, residues 98–102) and loop 5 (chain A, residues 299–314) together form a steric blockage which precludes binding sites at the minus end beyond –1, analogous to the steric barrier observed in *Cm*Man5A (residues 378–412); *(ii)* the substrate-binding region encompasses three glycosyl-binding subsites, *i*.*e*., subsite –1 and two product-binding sites +1 and +2. The residue His151 is suitably positioned to interact with one or both of the GlcNAc units, predicted to be located in subsites +1 and +2, to facilitate positioning of the substrate. In agreement with this function, His151 is conserved in *Cutibacterium* GH5_18 sequences as well as in other enzymes belonging to *Propionibacteriaceae*. We have used several experimental methods to show that *Ca*Man5_18 exists as a functional dimer in solution, a feature that appears to be uncommon for GH5 enzymes. In the crystal structure, important residues involved in dimer stabilization (Asp19, Asn52, Arg53, Arg57, Ser89, Asp91, Thr99, Trp100, His101, Gln143, His153 and Asp306) are conserved in all or most GH5_18 sequences (S4 Fig), suggesting that GH5_18 enzymes in general are likely to form functional dimers.

Prior to the intracellular deconstruction of the Man_3_-GlcNAc_2_ core, the full-length *N*-glycan antenna has to be trimmed on the extracellular side by secreted GH enzymes, and the resulting Man_3_-GlcNAc_2_ oligosaccharide must be transported across the plasma membrane. We could identify possible candidate genes for the extracellular pathway, but in contrast to the cytoplasmic pathway, the secreted GH genes were dispersed and did not map to any distinct gene cluster that could be assigned as a carbohydrate-processing locus. Gene candidates containing signal-peptide sequences were readily identified (Fig 3B): a *β*-1,2-*N*-acetylhexoaminidase gene (GH3; AEE73363), a *β*-1,4-galactosidase gene (GH35, AEE73032), and a potential sialidase gene (GH33; AEE72802).

The only gene that we have so far not been able to identify in the *C*. *acnes* genome is that coding for peptide-*N*-glycosidase F activity (PNGase F), *i*.*e*., the amidase responsible for hydrolyzing the amide bond to the host protein asparagine. As an alternative to PNGase-F activity, an endo-*β*-*N*-acetylglucosaminidase (GH18; AEE72208) would be able to cleave within the chitobiose core of *N*-linked glycans to release the glycan from the host protein as is the case for *S*. *pyogenes*, which produces two endoglycosidases, EndoS and EndoS2 that selectively hydrolyze Fc-bound *N*-glycans on therapeutic antibodies [14,69]. Another possibility is that another symbiotic bacterial skin colonizer provides the PNGase-F enzyme.

Although the phylotype 1A1 *C*. *acnes* strain 266 was isolated from a pleuropulmonary infection rather than skin [26], its NGD locus 1 (Fig 2) is representative for the clade-IA phylotype. It is interesting to note that the proposed NGD pathway is present only in IA strains, while appearing in a truncated version in the IB, IC, II and III phylotypes (S1 Fig). Phylotype IA1 strains dominate in follicles of Caucasian patients with acne, whereas IA1, IA2, IB and II display heterogeneous distributions on healthy skin [70]. The skin in patients with severe acne and healthy subjects show markedly different distributions of *C*. *acnes* phylotypes [71].

In acne sufferers, a homogeneous distribution of *C*. *acnes* phylotypes is observed, with 1A1 as the dominating phylotype, as well as an overall loss of phylotype diversity and absence of phylotype II [71]. Healthy subjects however, carried a more diverse composition of phylotypes with roughly equal distribution of 1A1 and II. In the same study, the distribution of clonal complexes (CC) was analyzed, and revealed that the CC18 subgroup was more prevalent in acne patients, whereas CC53 (phylotype II and K based on the SLST classification) dominated in healthy subjects.

The intact NGD locus 1 would enable IA strains to efficiently depolymerize and utilize all types of host *N*-glycans for nutrition. This is further supported by the presence of associated genes coding for ABC transporters. The ability to process host *N*-glycans is likely to present a selective advantage for IA strains over non-IA strains under conditions of limiting nutrient supply. The presence of a GH33 sialidase (AEE72802) indicates that the extracellular NGD pathway in *C*. *acnes* strain 266 may also contribute to virulence. The homologous GH33 enzyme produced by the *C*. *acnes* strain KPA171202 (96% sequence identity to AEE72802, was experimentally confirmed to be an exo-*α*-sialidase that cleaves off sialoglycoconjugates in human sebocyte cultures [72]. Nakatsuji and co-workers showed that the *C*. *acnes* KPA171202 sialidase increased host susceptibility to *C*. *acnes* cytotoxicity and adhesion, and furthermore, that mice immunized with the sialidase acquired protective immunity against *C*. *acnes* [72]. Beyond confirming a role of this sialidase in the degradation of sialoglycoconjugates, the study highlights the strategic importance of *C*. *acnes* enzymes that degrade host glycans for new treatment of acne vulgaris.

## Conclusions

The commensal bacterium *Cutibacterium acnes* is a natural habitant of the human skin microbiota, but under certain circumstances, *C*. *acnes* can turn pathogenic and cause acne vulgaris. We have mined the genome of *C*. *acnes* and identified a set of genes that are likely candidates for degradation of host *N*-glycans. We have also presented the biochemical and structural characterization of one enzyme in the proposed pathway, namely the exo-*β*-1,4-mannosidase that is able to catalyze hydrolysis of the *β*-1,4-glycosidic bond between the second GlcNAc and the first mannose residue in the canonical eukaryotic *N*-glycan core. This is the first reported enzymatic activity of subfamily GH5_18, and the first characterized GH5 enzyme likely to be involved in *N*-glycan degradation. Our results provide a valuable platform for further efforts to elucidate the complete set of activities of the proposed *C*. *acnes N*-glycan degradation pathway.

Based on our structural and functional characterization of *Ca*Man5_18, and whole-genome analysis, we propose a pathway for complete *N*-glycan degradation and utilization by *C*. *acnes* strain 266, results that offer new insights into the host-microbe interaction of this bacterium. We propose that this pathway allows the *C*. *acnes* to use host *N*-glycans as a means of nutrition, and possibly, also to contribute to virulence in susceptible individuals. The ability to degrade *N*-glycans would complement the demonstrated ability of *C*. *acnes* to disassemble *O*-glycans, which makes this bacterium well adapted to utilize a wide range of host glycans.

## Supporting information

S1 Fig Comparative genomics of N-glycan-processing locus 1. Genomic comparison of the *N*-glycan processing locus 1 in *C*. *acnes* using the comparative genomics platform Sybil (http://sybil.sourceforge.net). The *C*. *acnes* 266 strain was used as reference genome (region 1545000–1564000). A subset of genomes was selected to represent the different *C*. *acnes* phylogenetic groups (IA1, IA2, IB, II and III). The GH genes of each locus are highlighted. Predicted function of other genes is given in Fig 2.
(PDF)

S2 Fig Unrooted maximum likelihood tree for subfamily GH5_18. The tree was generated using PhyML and was based on 55 GH5_18 sequences. Numbers at the nodes are derived from the Approximate Likelihood-Ratio Test. Sequences from the *Bifidobacterium*, *Streptomyces* and *Cutibacterium* are indicated.
(PDF)

S3 Fig Sequence alignment of *Ca*Man5_18 from different *Cutibacterium acnes* phylotypes. Sequence alignment using Clustal Omega (https://www.ebi.ac.uk/Tools/msa/clustalo) highlighting the difference in amino-acid sequence of the different *C*. *acnes* phylotypes IA, IB, II and III strains. *Ca*Man5_18 (AEE72695) originates from the type-IA1 *C*. *acnes* strain 266. The figure was prepared with ESPript3 (http://espript.ibcp.fr/ESPript/ESPript/).
(PDF)

S4 Fig Sequence alignment of *Ca*Man5_18 and family GH5_18 homologs. Sequence alignment using Clustal Omega (https://www.ebi.ac.uk/Tools/msa/clustalo). Strictly conserved and highly conserved residues are highlighted with red boxes and red lettering, respectively. Secondary-structure elements are shown on top as spirals and arrows for *α*-helices and *β*-strands, respectively. Solid stars indicate the active-site residues that are conserved. The GenBank accession numbers are as follows: CaMan5_18 (AEE72695); CaGH5_18 from *Cutibacterium avidum* 44067 (AGJ77370); KfGH5_18 from *Kribbella flavida* DSM 17836 (ADB34475); BlGH5_18 from *Bifidobacterium longum* subsp. infantis 157F (BAJ71452); SkGH5_18 from *Sanguibacter keddieii* DSM 10542 (ACZ21418); ScGH5_18 from *Streptomyces coelicolor* A3(2) (CAB61915) and AhGH5_18 from *Arcanobacterium haemolyticum* DSM 20595 ADH91800). The figure was prepared with ESPript3 (http://espript.ibcp.fr/ESPript/ESPript/).
(PDF)

S5 Fig Unfolding profile and SEC analysis for *Ca*Man5_18. Analysis of oligomeric state. (A) ThermoFluor-derived melting curve for *Ca*Man_18 in 50 mM sodium phosphate (pH 7.5), and in the presence of the dye SYPRO Orange. Relative fluorescence units (RFU) were plotted against temperature. The protein unfolding curve is biphasic, which is consistent with two unfolding transitions. (B) Representation of the data in A by plotting the first derivative (*d*RFU)/*d*T of the raw data against temperature. Two *T*_m_ values were derived from this curve, 48.8°C and 58.0°C. (C) Overlay of the SEC chromatograms for the standard proteins (black lines) and of *Ca*Man5_18 (red line). Inset: Standard curve used to extrapolate the molecular weight (R^2^ = 0.9974). *Ca*Man5_18 elutes at a volume corresponding to a molecular weight of 98 kDa.
(PDF)

S6 Fig Hypothetical *C*. *acnes* 266 *N*-glycan-processing locus 2. Gene organization of a second putative *N*-glycan processing locus in the genome of *C*. *acnes* 266. GH genes predicted as mannosidases are colored green, and GH genes with predicted *N*-acetylhexosaminidase activity are blue. The GH4 gene with unknown activity is colored dark gray. Other associated genes are colored light gray and include: predicted sugar ABC transporter permease (PERM), transcriptional-regulator gene (REG), sugar ABC-transporter substrate-binding protein (SBP), hypothetical proteins (HP), HAD-family phosphatase involved in carbohydrate transport (PHO), and sugar kinase (ROK). Accession numbers (GenBank, or RefSeq when GenBank was not available) are shown below each gene.
(PDF)
