# Supplementary material for: Structural and biochemical characterization of the Cutibacterium acnes exo-β-1,4-mannosidase that targets the N-glycan core of host glycoproteins
Source: PLoS One. 2018 Sep 27;13(9):e0204703. doi: 10.1371/journal.pone.0204703 (PMC6160142; doi:10.1371/journal.pone.0204703)
Supplement: S1 Fig — Genomic comparison of the N-glycan processing locus 1 in C. acnes using the comparative genomics platform Sybil (http://sybil.sourceforge.net). The C. acnes 266 strain was used as reference genome (region 1545000–1564000). A subset of genomes was selected to represent the different C. acnes phylogenetic groups (IA1, IA2, IB, II and III). The GH genes of each locus are highlighted. Predicted function of other genes is given in Fig 2. (PDF) [file pone.0204703.s001.pdf]

S1 Fig. Comparative genomics of *N*-glycan-processing locus 1.

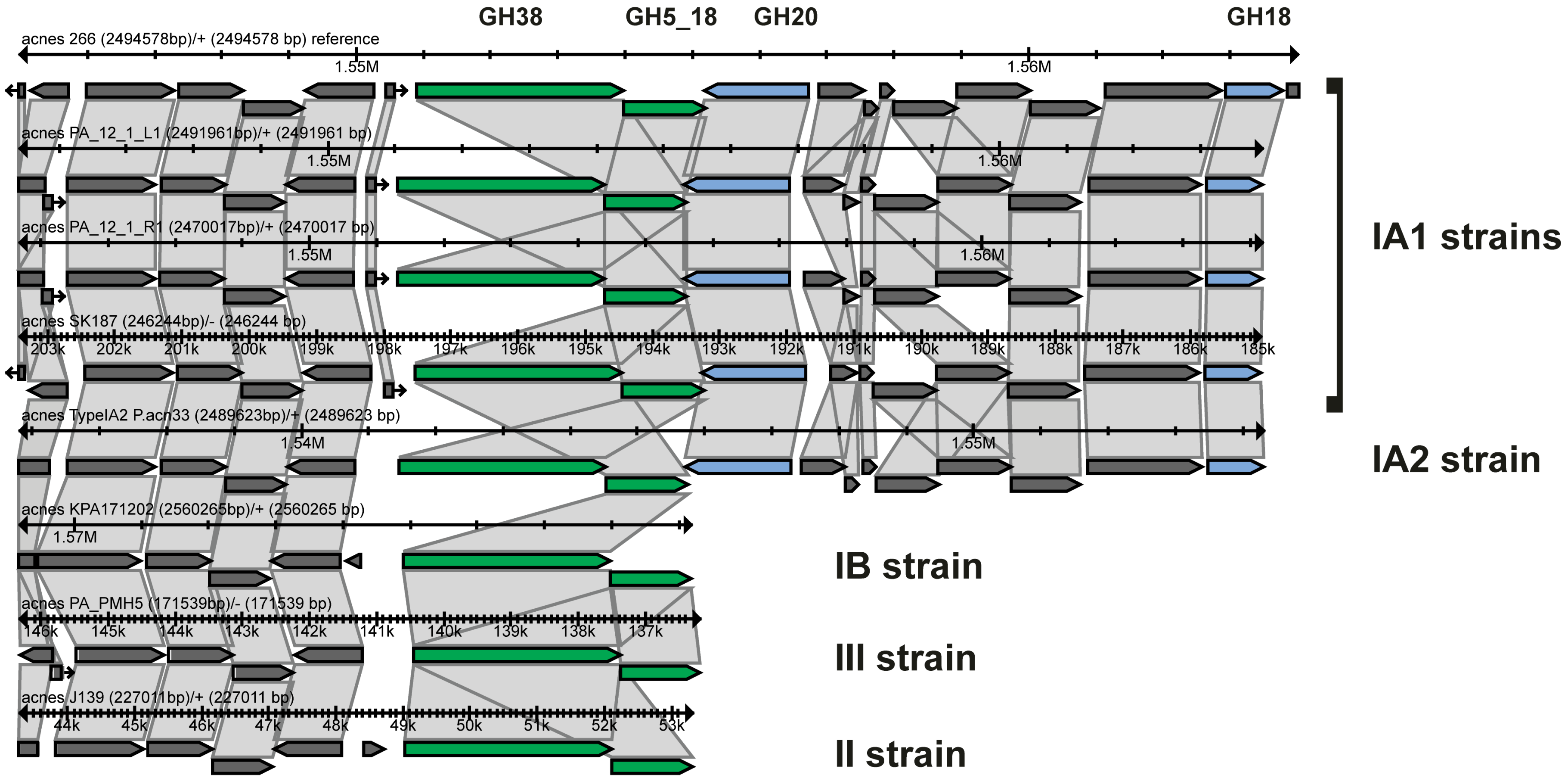

Genomic comparison of the *N*-glycan processing locus 1 in *C. acnes* using the comparative genomics platform Sybil (http://sybil.sourceforge.net). The *C. acnes* 266 strain was used as reference genome (region 1545000-1564000). A subset of genomes was selected to represent the different *C. acnes* phylogenetic groups (IA1, IA2, IB, II and III). The GH genes of each locus are highlighted. Predicted function of other genes is given in Figure 2.
