# Supplementary material for: Structural and biochemical characterization of the Cutibacterium acnes exo-β-1,4-mannosidase that targets the N-glycan core of host glycoproteins
Source: PLoS One. 2018 Sep 27;13(9):e0204703. doi: 10.1371/journal.pone.0204703 (PMC6160142; doi:10.1371/journal.pone.0204703)
Supplement: S2 Fig — The tree was generated using PhyML and was based on 55 GH5_18 sequences. Numbers at the nodes are derived from the Approximate Likelihood-Ratio Test. Sequences from the Bifidobacterium, Streptomyces and Cutibacterium are indicated. (PDF) [file pone.0204703.s002.pdf]

S2 Fig. Unrooted maximum likelihood tree for subfamily GH5_18.

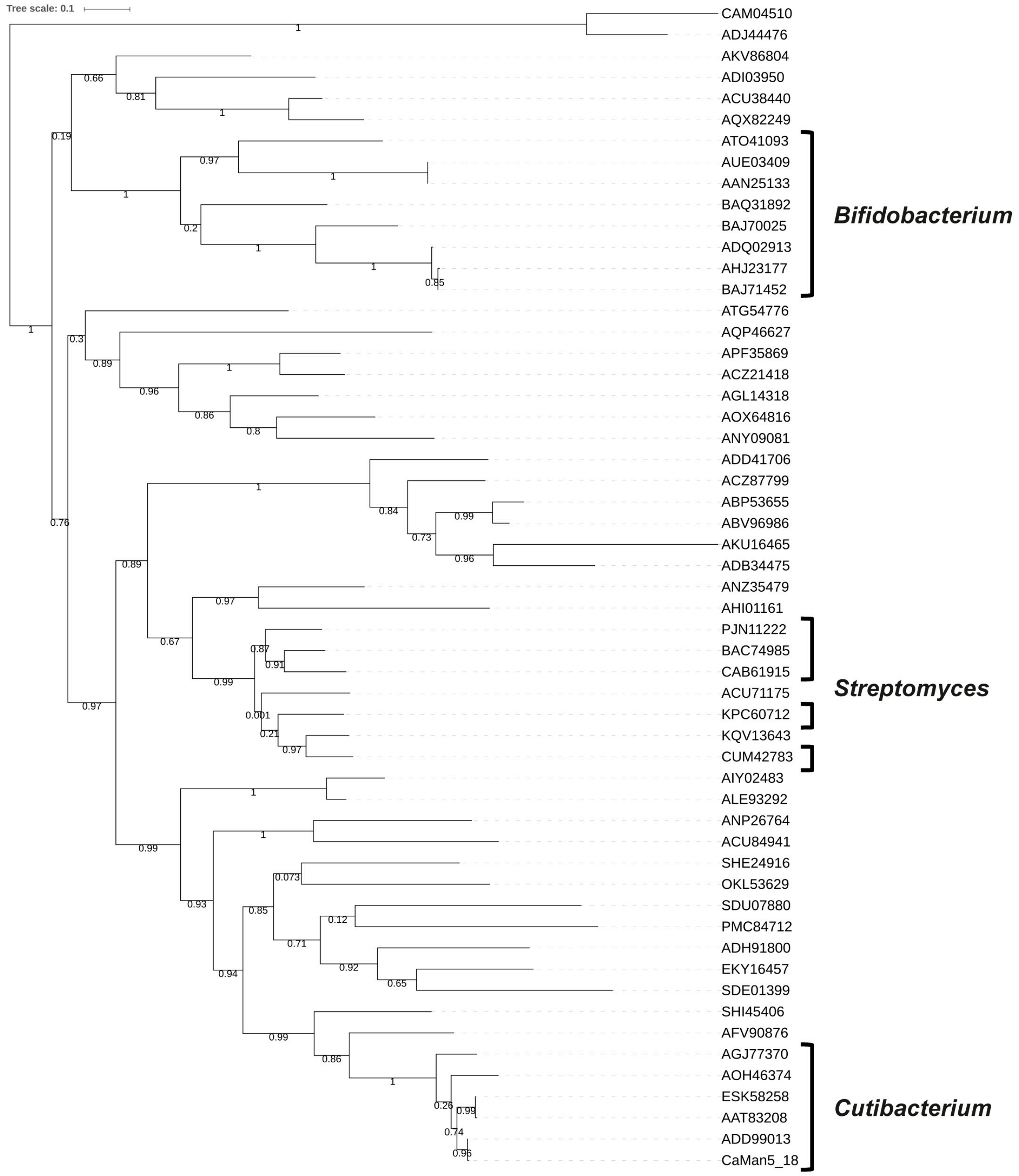

The tree was generated using PhyML and was based on 55 GH5_18 sequences. Numbers at the nodes are derived from the Approximate Likelihood-Ratio Test. Sequences from the *Bifidobacterium*, *Streptomyces* and *Cutibacterium* are indicated.
