# Supplementary material for: Structural and biochemical characterization of the Cutibacterium acnes exo-β-1,4-mannosidase that targets the N-glycan core of host glycoproteins
Source: PLoS One. 2018 Sep 27;13(9):e0204703. doi: 10.1371/journal.pone.0204703 (PMC6160142; doi:10.1371/journal.pone.0204703)
Supplement: S3 Fig — Sequence alignment using Clustal Omega (https://www.ebi.ac.uk/Tools/msa/clustalo) highlighting the difference in amino-acid sequence of the different C. acnes phylotypes IA, IB, II and III strains. CaMan5_18 (AEE72695) originates from the type-IA1 C. acnes strain 266. The figure was prepared with ESPript3 (http://espript.ibcp.fr/ESPript/ESPript/). (PDF) [file pone.0204703.s003.pdf]

S3 Fig. Sequence alignment of *Ca*Man5_18 from different *Cutibacterium acnes* phylotypes.

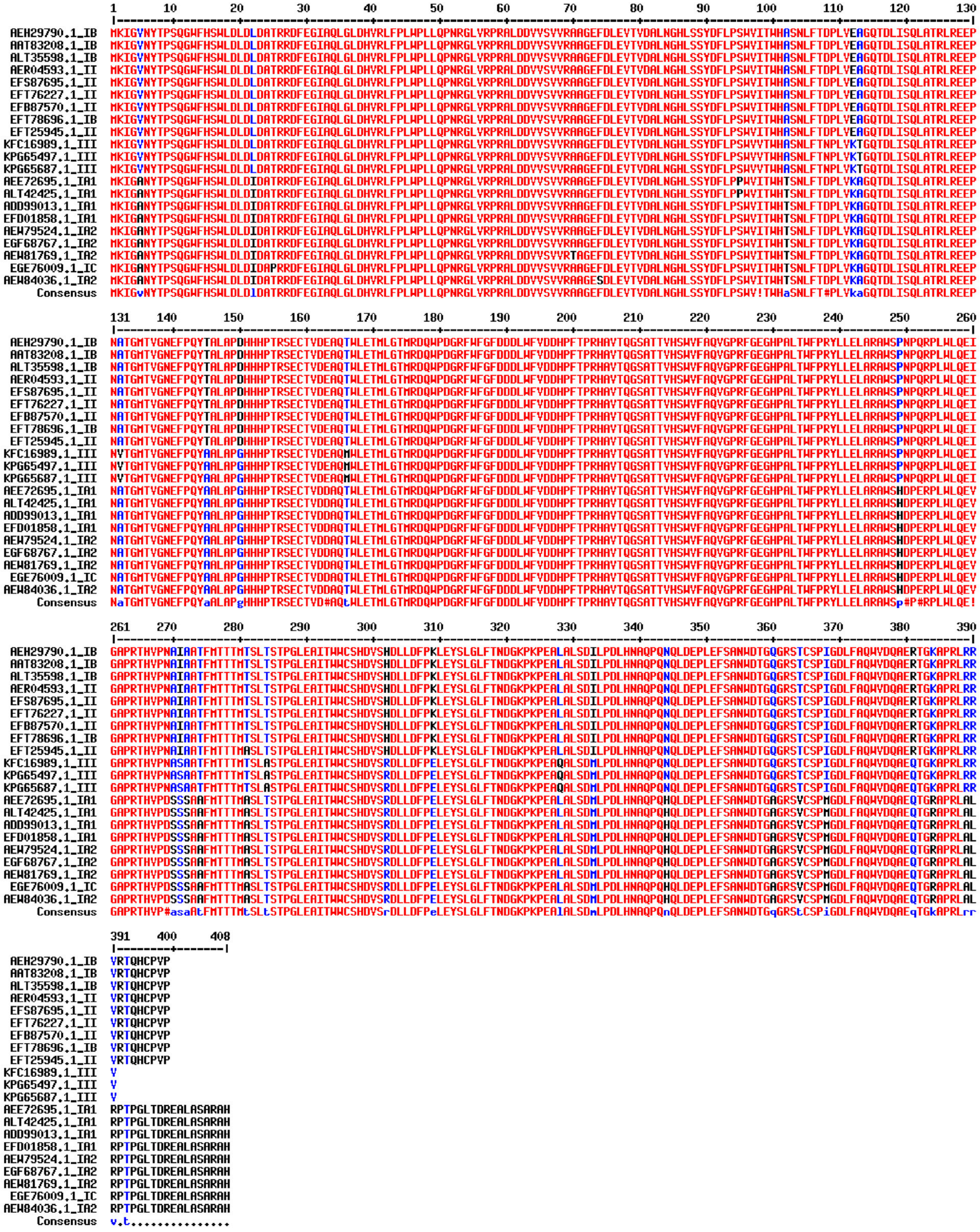

Sequence alignment using Clustal Omega (https://www.ebi.ac.uk/Tools/msa/clustalo) highlighting the difference in amino-acid sequence of the different *C. acnes* phylotypes IA, IB, II and III strains. *Ca*Man5_18 (AEE72695) originates from the type-IA1 *C. acnes* strain 266. The figure was prepared with ESPript3 (http://espript.ibcp.fr/ESPript/ESPript/).
