# Supplementary figures and images for: Structural and biochemical characterization of the Cutibacterium acnes exo-β-1,4-mannosidase that targets the N-glycan core of host glycoproteins
Source: PLoS One. 2018 Sep 27;13(9):e0204703. doi: 10.1371/journal.pone.0204703 (PMC6160142; doi:10.1371/journal.pone.0204703)

**S4 Fig. Sequence alignment of CaMan5\_18 and family GH5\_18 homologs.**

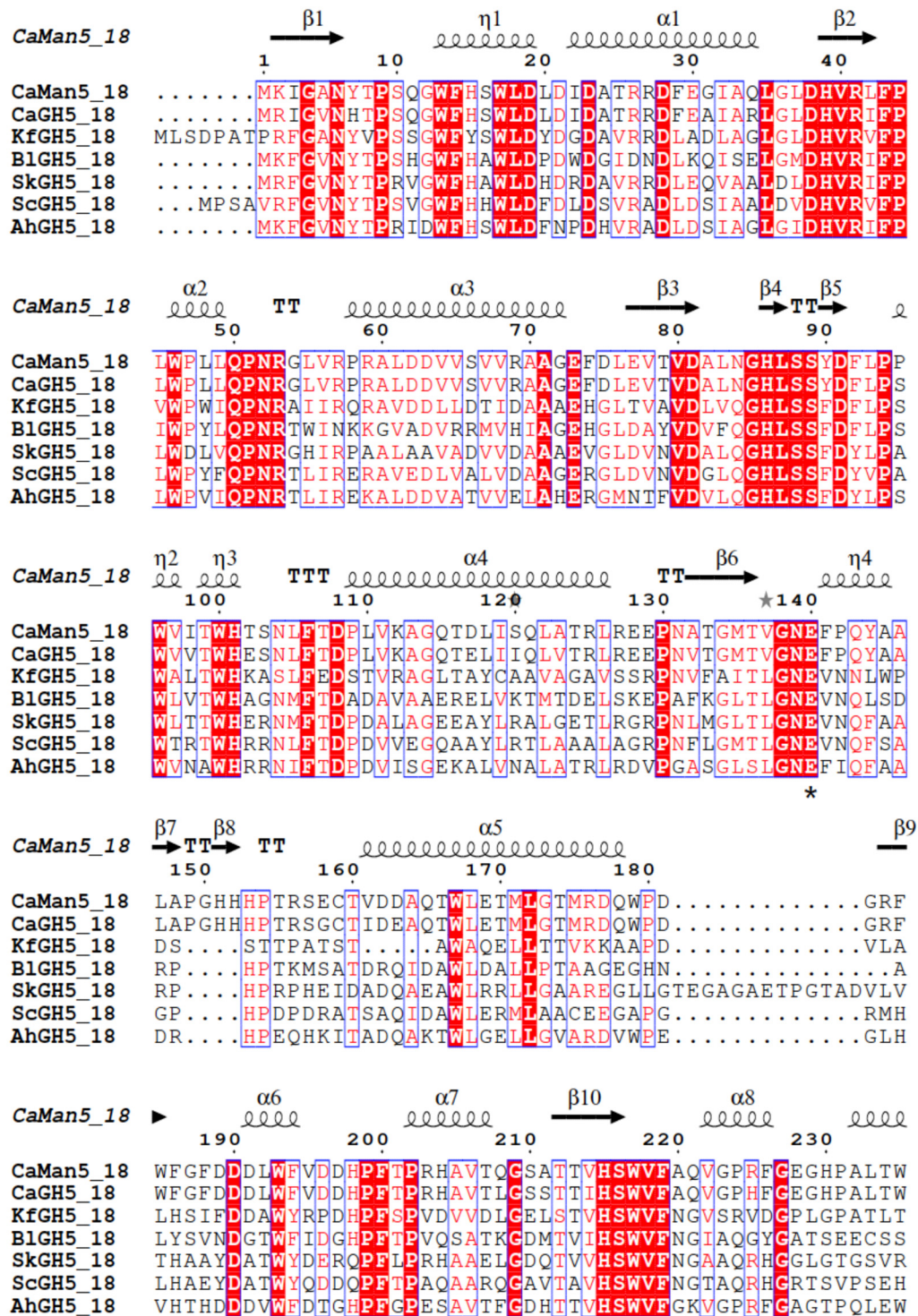

Supplement: S4 Fig — Sequence alignment using Clustal Omega (https://www.ebi.ac.uk/Tools/msa/clustalo). Strictly conserved and highly conserved residues are highlighted with red boxes and red lettering, respectively. Secondary-structure elements are shown on top as spirals and arrows for α-helices and β-strands, respectively. Solid stars indicate the active-site residues that are conserved. The GenBank accession numbers are as follows: CaMan5_18 (AEE72695); CaGH5_18 from Cutibacterium avidum 44067 (AGJ77370); KfGH5_18 from Kribbella flavida DSM 17836 (ADB34475); BlGH5_18 from Bifidobacterium longum subsp. infantis 157F (BAJ71452); SkGH5_18 from Sanguibacter keddieii DSM 10542 (ACZ21418); ScGH5_18 from Streptomyces coelicolor A3(2) (CAB61915) and AhGH5_18 from Arcanobacterium haemolyticum DSM 20595 ADH91800). The figure was prepared with ESPript3 (http://espript.ibcp.fr/ESPript/ESPript/). (PDF) [file pone.0204703.s004.pdf]
