# Supplementary material for: Structural and biochemical characterization of the Cutibacterium acnes exo-β-1,4-mannosidase that targets the N-glycan core of host glycoproteins
Source: PLoS One. 2018 Sep 27;13(9):e0204703. doi: 10.1371/journal.pone.0204703 (PMC6160142; doi:10.1371/journal.pone.0204703)
Supplement: S5 Fig — Analysis of oligomeric state. (A) ThermoFluor-derived melting curve for CaMan_18 in 50 mM sodium phosphate (pH 7.5), and in the presence of the dye SYPRO Orange. Relative fluorescence units (RFU) were plotted against temperature. The protein unfolding curve is biphasic, which is consistent with two unfolding transitions. (B) Representation of the data in A by plotting the first derivative (dRFU)/dT of the raw data against temperature. Two Tm values were derived from this curve, 48.8°C and 58.0°C. (C) Overlay of the SEC chromatograms for the standard proteins (black lines) and of CaMan5_18 (red line). Inset: Standard curve used to extrapolate the molecular weight (R2 = 0.9974). CaMan5_18 elutes at a volume corresponding to a molecular weight of 98 kDa. (PDF) [file pone.0204703.s005.pdf]

S5 Fig. Unfolding profile and SEC analysis for *Ca*Man5_18

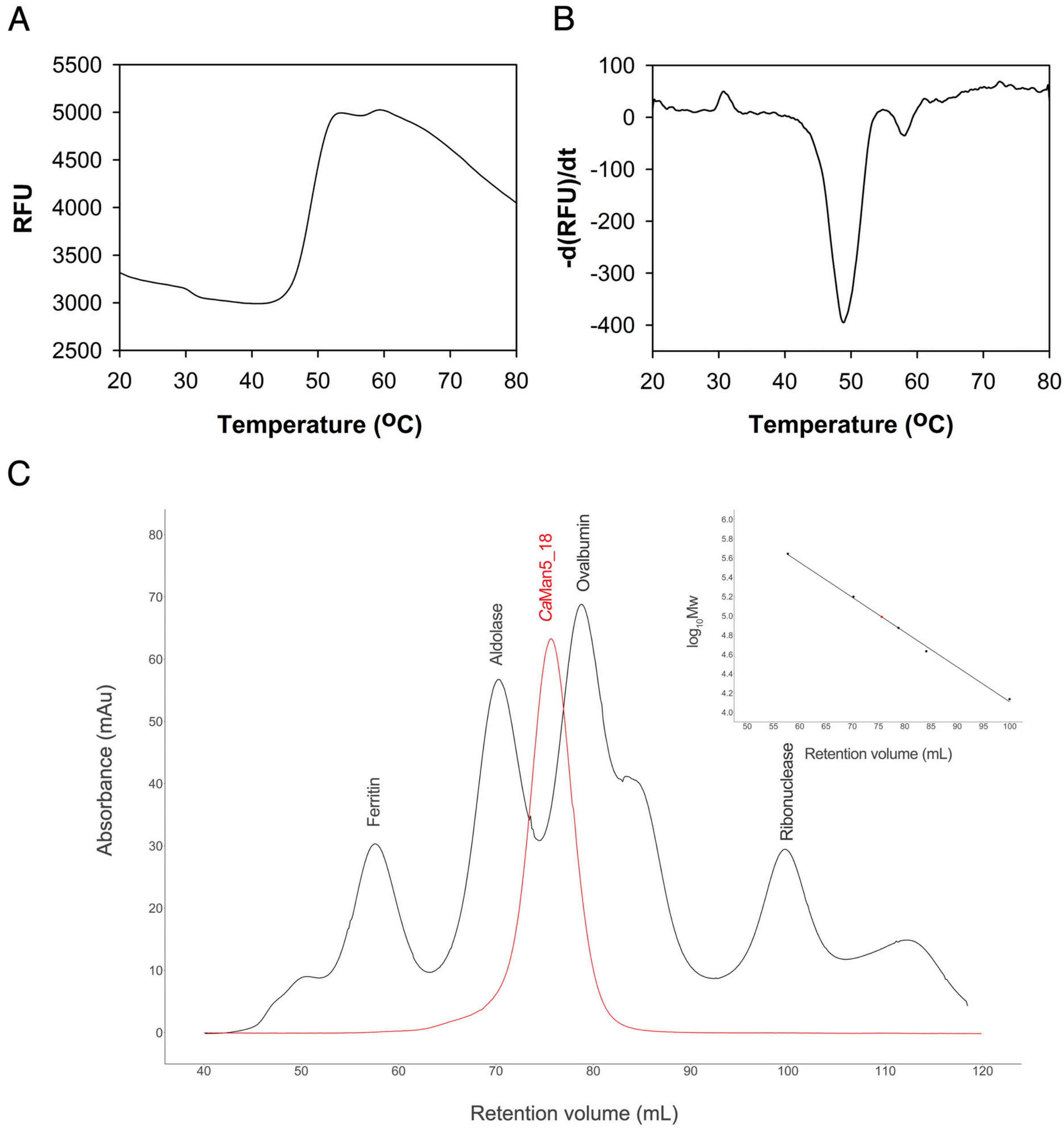

Analysis of oligomeric state. (A) ThermoFluor-derived melting curve for *Ca*Man_18 in 50 mM sodium phosphate (pH 7.5), and in the presence of the dye SYPRO Orange. Relative fluorescence units (RFU) were plotted against temperature. The protein unfolding curve is biphasic, which is consistent with two unfolding transitions. (B) Representation of the data in A by plotting the first derivative (*d*RFU)/*d*T of the raw data against temperature. Two T_m values were derived from this curve, 48.8°C and 58.0°C. (C) Overlay of the SEC chromatograms for the standard proteins (black lines) and of *Ca*Man5_18 (red line). Inset: Standard curve used to extrapolate the molecular weight (R^2=0.9974). *Ca*Man5_18 elutes at a volume corresponding to a molecular weight of 98 kDa.
