# Supplementary material for: Structural and biochemical characterization of the Cutibacterium acnes exo-β-1,4-mannosidase that targets the N-glycan core of host glycoproteins
Source: PLoS One. 2018 Sep 27;13(9):e0204703. doi: 10.1371/journal.pone.0204703 (PMC6160142; doi:10.1371/journal.pone.0204703)
Supplement: S6 Fig — Gene organization of a second putative N-glycan processing locus in the genome of C. acnes 266. GH genes predicted as mannosidases are colored green, and GH genes with predicted N-acetylhexosaminidase activity are blue. The GH4 gene with unknown activity is colored dark gray. Other associated genes are colored light gray and include: predicted sugar ABC transporter permease (PERM), transcriptional-regulator gene (REG), sugar ABC-transporter substrate-binding protein (SBP), hypothetical proteins (HP), HAD-family phosphatase involved in carbohydrate transport (PHO), and sugar kinase (ROK). Accession numbers (GenBank, or RefSeq when GenBank was not available) are shown below each gene. (PDF) [file pone.0204703.s006.pdf]

S6 Fig. Hypothetical *C. acnes* 266 *N*-glycan-processing locus 2.

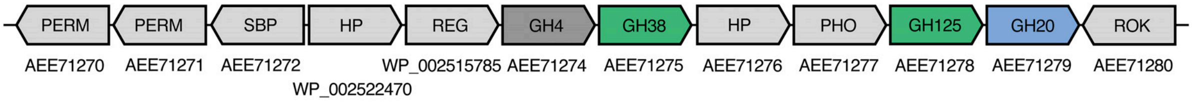

Gene organization of a second putative *N*-glycan processing locus in the genome of *C. acnes* 266. GH genes predicted as mannosidases are colored green, and GH genes with predicted *N*-acetylhexosaminidase activity are blue. The GH4 gene with unknown activity is colored dark gray. Other associated genes are colored light gray and include: predicted sugar ABC transporter permease (PERM), transcriptional-regulator gene (REG), sugar ABC-transporter substrate-binding protein (SBP), hypothetical proteins (HP), HAD-family phosphatase involved in carbohydrate transport (PHO), and sugar kinase (ROK). Accession numbers (GenBank, or RefSeq when GenBank was not available) are shown below each gene.
